# Phylogenetic turnover during subtropical forest succession across environmental and phylogenetic scales

**DOI:** 10.1002/ece3.3564

**Published:** 2017-11-15

**Authors:** Oliver Purschke, Stefan G. Michalski, Helge Bruelheide, Walter Durka

**Affiliations:** ^1^ German Centre for Integrative Biodiversity Research (iDiv) Halle‐Jena‐Leipzig Leipzig Germany; ^2^ Geobotany and Botanical Garden Institute of Biology Martin Luther University Halle‐Wittenberg Halle (Saale) Germany; ^3^ Department of Community Ecology Helmholtz Centre for Environmental Research – UFZ Halle (Saale) Germany

**Keywords:** chronosequence, community assembly, depth of turnover, environmental filtering, null model, phylogenetic niche conservatism

## Abstract

Although spatial and temporal patterns of phylogenetic community structure during succession are inherently interlinked and assembly processes vary with environmental and phylogenetic scales, successional studies of community assembly have yet to integrate spatial and temporal components of community structure, while accounting for scaling issues. To gain insight into the processes that generate biodiversity after disturbance, we combine analyses of spatial and temporal phylogenetic turnover across phylogenetic scales, accounting for covariation with environmental differences. We compared phylogenetic turnover, at the species‐ and individual‐level, within and between five successional stages, representing woody plant communities in a subtropical forest chronosequence. We decomposed turnover at different phylogenetic depths and assessed its covariation with between‐plot abiotic differences. Phylogenetic turnover between stages was low relative to species turnover and was not explained by abiotic differences. However, within the late‐successional stages, there was high presence‐/absence‐based turnover (clustering) that occurred deep in the phylogeny and covaried with environmental differentiation. Our results support a deterministic model of community assembly where (i) phylogenetic composition is constrained through successional time, but (ii) toward late succession, species sorting into preferred habitats according to niche traits that are conserved deep in phylogeny, becomes increasingly important.

## 1 INTRODUCTION

A better understanding of the processes that generate biodiversity during succession after disturbance is needed for more accurate predictions of ecosystem responses to future disturbance events (Dornelas, 2010; Garnier et al., 2004). Community assembly during succession may be driven by deterministic (biotic and abiotic filtering) as well as stochastic processes (Fukami, Martijn Bezemer, Mortimer, & van der Putten, 2005; Keddy, 1992) that are often inferred using trait‐based approaches (Bazzaz, 1979; Shipley, Vile, & Garnier, 2006). However, the traits involved in assembly processes are *a priori* unknown and, particularly in species‐rich systems, it is difficult to choose and measure the most relevant traits. In communities with broad taxonomic sampling, such as hyper‐diverse tropical plant communities, closely related species often share similar functional characteristics (Swenson, 2013), resulting from phylogenetic niche conservatism (Losos, 2008). In such systems, phylogenetic relatedness between species is often used as a proxy for overall trait similarity as it potentially integrates more trait information than a limited set of measurable traits (Mouquet et al., 2012; Pavoine & Bonsall, 2011). Several studies have quantified spatial or temporal patterns of phylogenetic relatedness throughout succession, either by testing for nonrandom patterns of relatedness within‐successional stages (Ding, Zang, Letcher, Liu, & He, 2012; Letcher, 2010) or by examining whether the observed temporal phylogenetic turnover between stages differed from the expected phylogenetic turnover, given the level of species turnover (Letten, Keith, & Tozer, 2014; Swenson et al., 2012). However, purely temporal approaches, that focus on phylogenetic turnover *between* stages, do not allow to evaluate whether nonrandom patterns of temporal phylogenetic turnover are simply a reflection of spatial turnover between sites belonging to the same successional stage (see Purschke et al., 2013). In contrast, approaches that focus on spatial patterns of phylogenetic relatedness *within* successional stages only allow for inferences about assembly processes that act at a particular successional stage. Because spatial and temporal patterns of community composition are inherently interlinked (Preston, 1960; White, Ernest, Adler, Hurlbert, & Lyons, 2010), studies based on partial analysis of either spatial or temporal patterns of community phylogenetic structure during succession will only give limited insight into the temporal dynamics of assembly processes.

Hardy and Senterre (2007) proposed a framework that allows to test the spatial phylogenetic structure of communities, based on the extent to which species within sites are more, or less, related to each other than to species from different sites. If species that co‐occur within a site are more related to each other than to species from different sites, phylogenetic turnover between sites is high, which is referred to as spatial phylogenetic clustering. Such high phylogenetic turnover is usually interpreted as a signature of abiotic filtering where distinct groups of closely related, and functionally similar, species are differentially selected in sites that differ in their environmental conditions (Baraloto et al., 2012). Alternatively, phylogenetic clustering may reflect the exclusion of competitively inferior species, resulting from competitive hierarchies, if the traits conferring competitive dominance are phylogenetically conserved (Mayfield & Levine, 2010). In contrast, if species within sites are phylogenetically less related than species from different sites, phylogenetic turnover between sites is low, which is referred to as spatial phylogenetic overdispersion. This pattern is often interpreted as result of biotic filtering because of negative interactions due to limiting similarity competition between closely related species, but could also indicate abiotic filtering in case of convergent evolution of important niche traits (Cavender‐Bares, Ackerly, Baum, & Bazzaz, 2004). Because the Hardy and Senterre (2007) framework expresses community differentiation between sites, it can also be applied to pairs of communities at different successional stages (see Purschke et al., 2013), allowing to compare spatial and temporal patterns of community differentiation within a consistent framework.

Despite the promise of combining spatial and temporal components of phylogenetic turnover to gain insight into assembly processes, there remain several difficulties with interpreting community phylogenetic structure. One main problem is that patterns of phylogenetic relatedness within communities and conclusions about assembly processes are highly scale‐dependent (Graham, Macháč, & Storch, 2016; Swenson, Enquist, Thompson, & Zimmerman, 2007). For instance, patterns of phylogenetic overdispersion will only be detectable at small environmental, spatial, and phylogenetic scales (i.e., between closely related species close to tips of the phylogeny, see Parmentier et al., 2014). In contrast, phylogenetic clustering, resulting from abiotic filtering, has mainly been demonstrated over steep to moderate ecological gradients and at large phylogenetic scales deep in the phylogeny (Cavender‐Bares, Keen, & Miles, 2006). In addition, Hardy and Senterre (2007) pointed out that if such opposing assembly mechanisms, like overdispersion and clustering, act simultaneously at different phylogenetic scales, they may cancel out each other, resulting in an overall random phylogenetic structure. To address this phylogenetic scaling issue, phylogenetic structure can be assessed at different depths in the phylogenetic tree (Cavender‐Bares & Reich, 2012; Hardy & Senterre, 2007). The issue of environmental scaling may be accounted for by assessing the extent to which phylogenetic turnover is explained by environmental differences between sites (e.g., Hardy, Couteron, Munoz, Ramesh, & Pélissier, 2012).

Finally, inferences about assembly processes may be influenced by the level of biological organization considered in the analysis, i.e. whether phylogenetic structure is assessed on the level of species or individuals, respectively, giving more weight to rare or dominant species (Helmus, Bland, Williams, & Ives, 2007; Lozupone, Hamady, Kelley, & Knight, 2007). The joint use of abundance‐ and presence‐/absence‐based indices allows to detect the relative importance of shifts in species abundances vs. changes in composition, and hence will be critical to understand the processes underlying community assembly (Vellend, Cornwell, Magnuson‐Ford, & Mooers, 2011).

In the context of succession, theory predicts that in early succession, disturbance acts as an environmental filter selecting for closely related species and that biotic filtering will become more important over time, selecting for more distantly related species in late succession (Connell & Slatyer, 1977). While a number of studies found support for this hypothesis (e.g., Letcher, 2010; Whitfeld et al., 2012; Purschke et al., 2013), a few recent studies detected an increase in phylogenetic relatedness during succession, and suggested that hierarchical competition and/or environmental filtering become more important during succession (e.g., Buzzard, Hulshof, Birt, Violle, & Enquist, 2016; Kunstler et al., 2012; Letten et al., 2014; Uriarte et al., 2010). However, existing studies of phylogenetic community structure (i) were usually based on metrics of phylogenetic structure that integrate across the whole phylogeny, and therefore did not allow for the possibility that assembly processes will only be detectable at particular phylogenetic scales, (ii) did not include information on environmental differentiation between sites, or (iii) focused either on spatial or temporal components of community change. To gain more accurate insights into the processes that underlie community assembly during succession after disturbance, there is therefore a need for integrative studies that account for phylogenetic community structure at different phylogenetic scales and that compare spatial and temporal turnover components in conjunction with environmental differentiation between sites. If, for example, abiotic filtering along an environmental gradient is the predominant process shaping communities at the beginning of succession, and there is phylogenetic conservatism in species’ traits conferring their environmental tolerances, spatial phylogenetic turnover between early‐successional communities will (i) be higher than expected given the level of species turnover, (ii) be explained by environmental differences between communities (Bartlett et al., 2016; Cadotte & Tucker, 2017) and iii) be detected only at large phylogenetic scales (Cavender‐Bares & Reich, 2012; Hardy et al., 2012). If, in contrast, there is an increase in the relative importance of biotic filtering, due to limiting similarity competition, during succession, we predict that spatial phylogenetic turnover between late‐successional communities will be (i) less than expected (spatial phylogenetic overdispersion), (ii) detected at small phylogenetic scales, and (iii) unrelated to environmental differences between plots (Bartlett et al., 2016). Alternatively, if hierarchical competition is the predominant force shaping communities during late succession, we predict that late‐successional communities will be comprised of closely related species but that phylogenetic turnover will not covary with environmental differentiation (Bartlett et al., 2016). If traits conferring competitive dominance are phylogenetically conserved, and competitively superior species belong to a particular clade (Roeder et al., 2015), we additionally predict that hierarchical competition will cause phylogenetic clustering at shallow phylogenetic scales. In contrast, if late‐successional communities are primarily governed by the accumulation of closely related species that share adaptations to the local abiotic conditions (Li et al., 2015) and environmental filtering selects for distinct sets of closely related species in plots that differ in their abiotic environment, we predict that spatial phylogenetic turnover between communities belonging to the late‐successional stages will be (i) higher than expected, (ii) explained by environmental differences between sites, and (iii) detected at broad phylogenetic scales, resulting from phylogenetic conservatism of abiotic niches. Finally, if deterministic community assembly results in temporal shifts in phylogenetic community composition due to successional changes in abiotic conditions (Swenson et al., 2012), we predict that phylogenetic turnover between stages will (i) be higher than expected by chance, (ii) be higher than spatial turnover between plots from the same stage, and (iii) increase with environmental differences between stages. Conversely, if relatively constant abiotic conditions cause a lack of phylogenetic shifts to over time (Letten et al., 2014), we predict that phylogenetic turnover between successional stages will be (i) low relative to species turnover, (ii) lower than phylogenetic turnover between plots from the same stage, and (iii) unrelated to environmental differences between stages.

To test these predictions, we use data on tree communities representing different stages of a subtropical forest succession in south‐eastern China. Successional subtropical forests provide an ideal system for the study of temporal changes in the mechanisms underlying community assembly as they represent community assembly in action and are exceptionally species‐rich (Arroyo‐Rodríguez et al., 2017; Uriarte et al., 2010). While subtropical forest areas were once widespread across South and East China, they are currently under severe decline as a result of land use intensification (Wang, Kent, & Fang, 2007). Because of frequent anthropogenic disturbance events, such as logging and burning, subtropical forests often consist of a mosaic of different stages of secondary forest succession. Combining analysis of spatial and temporal turnover (at the individual‐ and species‐level), while examining turnover (i) at different phylogenetic depths and (ii) with increasing environmental differentiation, we will be able to address competing predictions about the temporal changes in the relative importance of the processes that generate biodiversity after disturbance.

## 2 MATERIALS AND METHODS

### 2.1 Study area and sampling

We studied woody plant communities in the comparative study plots that had been established within the biodiversity–ecosystem functioning experiment BEF‐China (Bruelheide et al., 2011). The plots represent a chronosequence of subtropical forest succession in the Gutianshan National Nature Reserve (GNNR), located in Zhejiang Province in south‐eastern China (29°8′18′’–29°17′29′’ N, 118°2′14′’–118°11′12′’ E). The GNNR comprises mixed broad‐leaved forests (Hu & Yu, 2008; Wu, 1980) within an elevational range of 250 m to 1258 m a.s.l.. A total of 1426 seed plant species of 648 genera and 149 families has been recorded in GNNR (Lou & Li, 1988). The study area mainly consists of a mosaic of secondary forest stands that represent different successional stages, with maximum tree age of approximately 180 years (Bruelheide et al., 2011).

Species abundance data were obtained from a vegetation inventory (May–October 2008) of all individuals of trees and shrubs (>1 m height, 147 species in total) in each of the 27 30 × 30 m plots (see Bruelheide et al., 2011). The plots were distributed over the GNNR to represent five successional stages (differing by 20 years), based on estimations of the age of the largest tree individuals and on knowledge of the last logging event [see Bruelheide et al. *(* 2011) for more detailed information on type of disturbance that preceded succession]. The number of plots per successional stage was 5 (<20 years), 4 (20–39 years), 5 (40–59 years), 6 (60–79 years), and 7 (≥80 years). Because fewer individuals were recorded in the older plots relative to the younger plots (Fig. S1), we assessed whether the differences in the number of individuals between plots may potentially bias our results, which was not the case in our study (Table S1).

For each plot, a set of environmental variables (Table S2) related to topography [aspect (expressed as northness and eastness), slope, elevation], light (photosynthetically active radiation (PAR), red/far‐red ratio) and soil characteristics (pH, moisture, C/N‐ratio) were available from Bruelheide et al. (2011) and Kröber, Böhnke, Welk, Wirth, and Bruelheide (2012). Total phosphorus (P) content of the soil was measured with nitric acid digestion, a standard method recommended by the German forest soil survey (BMELV, 2009). The inorganic nitrogen concentration (NO3^‐^, NH4^+^) of the mineral soil was determined by KCl extraction (1 mol/L) followed by Flow Injection Analysis (FIAstar 500 Analyzer, FOSS, Hilerød, Denmark).

### 2.2 Phylogenetic data and regional species pool

Based on the set of species present in the 27 plots and on the list of all woody species of the Gutianshan National Nature Reserve (Lou & Li, 1988), we constructed a regional species pool [the set of 438 woody species that occur in the whole GNNR (Table S3)] for which a phylogeny was inferred. For details on phylogenetic inference see Methods S1 and Tables S4 & S5. In short, we obtained sequence information (matK, rbcL, and ITS region) for all species, or their closest relatives, from GenBank or de novo using standard barcoding protocols. A maximum‐likelihood tree was computed and dated using nonparametric rate smoothing and using published fossils as age constraints (Methods S2 and S3). To avoid potential bias in the analysis of phylogenetic patterns due to their disproportionately long‐branch lengths (Cadotte, 2014; Letcher, 2010), nonangiosperm, and one bamboo (*Pleioblastus amarus,* Poaceae) species, which generally occurred at low frequencies within the study area, were excluded from the regional species pool. We further excluded cultivated species, resulting in a total of 410 woody species of which 143 occurred in the 27 study plots (Table S3).

### 2.3 Phylogenetic structure

Using information on species composition and the phylogenetic tree pruned down to the 143 woody angiosperms found in the 27 plots, we estimated phylogenetic structure following the framework proposed by Hardy and Senterre (2007), which is based on the spatial decomposition of evolutionary relatedness between species into within‐ and between‐community components. Within the Hardy and Senterre (2007) framework, spatial phylogenetic structure was quantified for presence/absence and abundance data, using the phylogenetic turnover (between‐plot differentiation) statistics Π_ST_ and B_ST_, respectively: Π_ST_ = 1 – Δ^P^_w_/Δ^P^_a_ and B_ST_ = 1 – Δ*^P^_w_/Δ*^P^_a_, where Δ^P^_w_ and Δ*^P^_w_ represent phylogenetic alpha diversity, and correspond to the mean within‐community phylogenetic distance between distinct species and the mean phylogenetic distance between two individuals of distinct species, respectively, averaged over all communities belonging to the same successional stage. Δ^P^_a_ and Δ*^P^_a_ are the mean phylogenetic distance between distinct species and the mean phylogenetic distance between two individuals of distinct species, respectively, sampled from different communities belonging to a particular stage. Values of spatial phylogenetic turnover, Π_ST_ or B_ST_, > 0 indicate spatial phylogenetic clustering—species, or individuals, within communities are phylogenetically more related than species, or individuals, from different communities. Spatial phylogenetic overdispersion is observed if Π_ST_ or B_ST_ < 0, indicating that species, or individuals, within communities are phylogenetically less related than species, or individuals, from different communities. When Π_ST_ and B_ST_ are calculated between pairs of plots belonging to the same successional stage, they address within‐stage phylogenetic turnover. When Π_ST_ and B_ST_ are calculated between pairs of plots belonging to different successional stages, they address between‐stage phylogenetic turnover. We tested, based on 100 simulation runs, whether levels of spatial phylogenetic turnover were affected by differences in the number of plots among stages (Methods S4). Mean Pearson correlations between Π_ST_ (or B_ST_) for simulated communities and the number plots were close to zero, indicating that levels of phylogenetic turnover were not simply a reflection of the number of plots. To complement our main analyses of phylogenetic turnover, and in addition to measures of phylogenetic alpha diversity (Δ^P^_w_ and Δ*^P^_w_), we also calculated Shannon evenness (Magurran, 2004) for each plot.

### 2.4 Null models

To test whether Π_ST_ or B_ST_ were significantly higher (or less) than zero, observed Π_ST_‐ or B_ST_‐values were compared to those recalculated for 999 random communities. Random communities were generated using null model “1p” in Hardy (2008), shuffling species names across the phylogeny of all 410 woody angiosperms from the regional species pool. The latter corresponding to the set of species that are present in, or could potentially colonize, our study plots (see Ding et al., 2012 and Letcher et al., 2012). This null model maintains (i) the number of species within each community, (ii) species turnover between communities, (iii) the patterns of spatial autocorrelation in overall species abundances and occurrence frequencies, (iv) species’ occurrence frequency across the study landscape, and (v) species identity within each successional time step. This type of null model is appropriate for temporal data (Letcher et al., 2012; Norden, Letcher, Boukili, Swenson, & Chazdon, 2012) and has been demonstrated to provide exact tests (i.e., correct Type‐I error rates) in situations where overall species frequencies (or abundances) are not phylogenetically structured (Hardy, 2008; see Methods S5). Significant positive (or negative) values of Π_ST_ (or B_ST_) of within‐stage phylogenetic turnover indicate that species, or individuals, co‐occurring within‐successional stages are more (or less) related than expected by chance. Higher‐than‐expected Π_ST_ or B_ST_ values of between‐stage phylogenetic turnover that are higher than within‐stage phylogenetic turnover indicate phylogenetic shifts during the course of succession. Lower‐than‐expected values of between‐stage turnover, that are lower than within‐stage turnover, would indicate constant phylogenetic composition during succession.

### 2.5 Phylogenetic structure at different depths in the phylogeny

We assessed whether nonrandom phylogenetic structure, within each of the five successional stages, occurred at particular phylogenetic depths, following the approach in Hardy and Senterre (2007): Phylogenetic turnover between plots was calculated based only on species pairs within clades younger than a given divergence time threshold. We chose eleven age thresholds, ranging between 30 Myr and 128 Myr, by steps of approximately 10 Myr. To test whether phylogenetic turnover significantly differed from zero at particular phylogenetic scales, we carried out partial randomizations, shuffling species names across the phylogeny, but restricting the randomization to species within clades younger than the respective age threshold. All calculations of phylogenetic community structure were carried out on phylogenetic, cophenetic distance matrices, using the packages “vegan” (Oksanen et al., 2017) and “spacodiR” (Eastman, Paine, & Hardy, 2011) in the R statistical package (R Development Core Team, 2017) and SPACoDi 0.10 (Hardy, 2010). To identify clades that significantly contributed to phylogenetic turnover between plots, we tested for each node in the phylogeny whether it had more descendent taxa than expected in a particular plot, using the “nodesig” procedure in Phylocom v.4.2 (Webb, Ackerly, & Kembel, 2009).

### 2.6 Relating phylogenetic structure to environmental variables

To quantify the extent to which spatial and temporal phylogenetic turnover was explained by differences in abiotic conditions, pairwise Π_ST_ (or B_ST_) values were regressed on between‐plot environmental distances. To control for covariation between phylogenetic turnover and spatial distance, we used the residuals from regressions of Π_ST_ (or B_ST_) against the Euclidean distances calculated from the geographic x‐ and y‐coordinates of the plots instead of the actual phylogenetic turnover values. Significance of the relationships was assessed by nonparametric randomization testing [5000 randomizations, R‐package “lmPerm” (Wheeler & Torchiano, 2016)]. Environmental distances were obtained from an interplot distance matrix based on the 11 topographic, light, and edaphic descriptors. A principal components analysis (PCA) was carried out on the log‐transformed and standardized (mean = 0, SD = 1) environmental data, to correct for the dominance of the distance matrix by highly correlated environmental variables. The resulting first six principal components (PCs) accounted for about 90% of the total variation (Table S6) and were used to construct the Euclidean interplot distance matrix from which the environmental distances were obtained. Because associations between phylogenetic turnover and environmental differentiation may be a reflection of differences in sample size among the successional stages, we additionally assessed relationships between environmental and phylogenetic turnover at each stage‐based resampling all possible combinations of four plots, the minimum number of plots across stages.

### 2.7 Phylogenetic signal in traits

To assess whether phylogenetic relatedness between species reflects their ecological similarity, we quantified phylogenetic signal in six traits [leaf area, specific leaf area (SLA), leaf nitrogen content, leaf phosphorus content, wood density, and maximum height] that represent multiple axes of plant functional differentiation (Chave et al., 2009; Moles et al., 2009; Westoby, Falster, Moles, Vesk, & Wright, 2002; Wright et al., 2004). Estimates of phylogenetic signal were based on the three metrics Blomberg’ *K* (Blomberg, Garland, & Ives, 2003), Pagel's λ (Pagel, 1999), and Abouheif/Moran's *I* (Abouheif, 1999) (Table S7), and calculated in the R‐packages “phytools” (Revell, 2012) and “adephylo” (Jombart, Balloux, & Dray, 2010), for the subset of 121 species (of the 143 angiosperm species occurring in the 27 plots) for which data on all six traits were available from Kröber et al. (2012), Böhnke, Kreißig, Kröber, Fang, and Bruelheide (2012), and Böhnke, Kröber, Welk, Wirth, and Bruelheide (2014).

## 3 RESULTS

### 3.1 Temporal changes in alpha diversity

**Figure 1:**
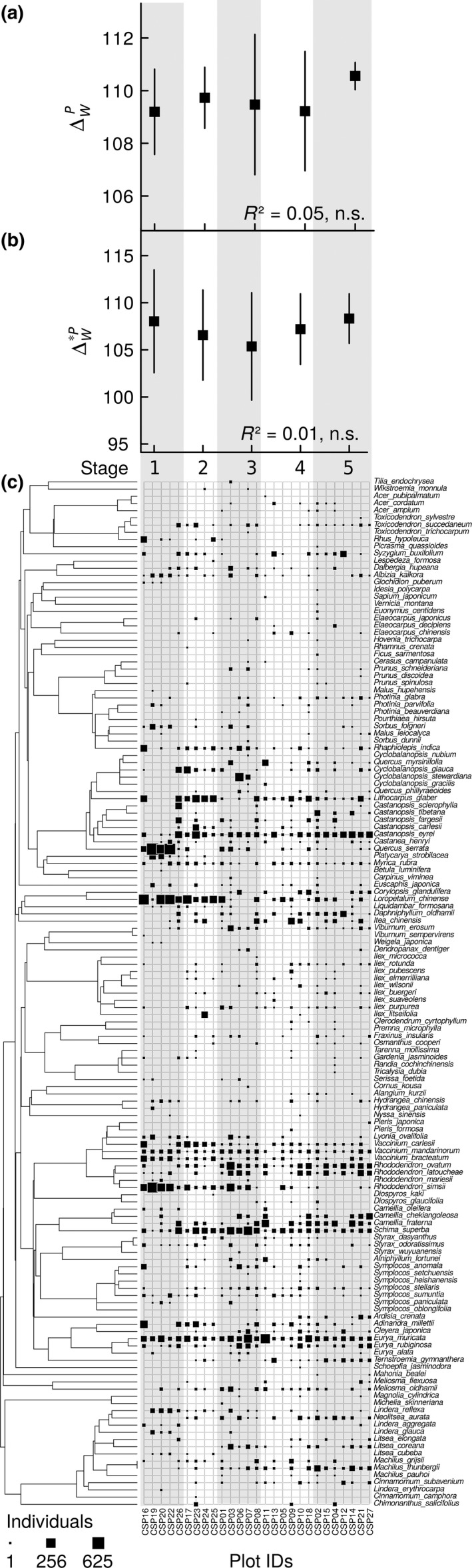
Phylogenetic alpha diversity within the five successional stages (mean ± 1 SE; Stage 1 (<20 years): *n *= 5, Stage 2 (20–39 years): *n *= 4, Stage 3 (40–59 years): *n *= 5, Stage 4 (60–79 years): *n *= 6, Stage 5 (≥80 years): *n *= 7), based on (a) presence/absence (Δ^*P*^_*w*_) and (b) abundance data (Δ^*P^_*w*_). Δ^*P*^_*w*_ and Δ^*P^_*w*_ are equivalent to the mean phylogenetic distance between distinct species (Δ^*P*^_*w*_), and the mean phylogenetic distance between individuals of distinct species (Δ^*P^_*w*_) within communities, respectively. *R* ^2^ values are given. None of the two alpha diversity measures showed a significant successional trend. (c) Distribution of abundances within the 27 comparative study plots [assigned to one of the five successional stages (Stage 1–5)] mapped onto the phylogeny of the 143 species. The size of the black squares corresponds to the number of individuals

Phylogenetic alpha diversity (Δ^P^_w_ and Δ*^P^_w_) showed no significant temporal trend during the course of succession (Figure 1a,b). In contrast, there was a steep increase in species (Shannon) evenness over time (Fig. S2).

### 3.2 Comparisons between spatial and temporal phylogenetic turnover

**Figure 2:**
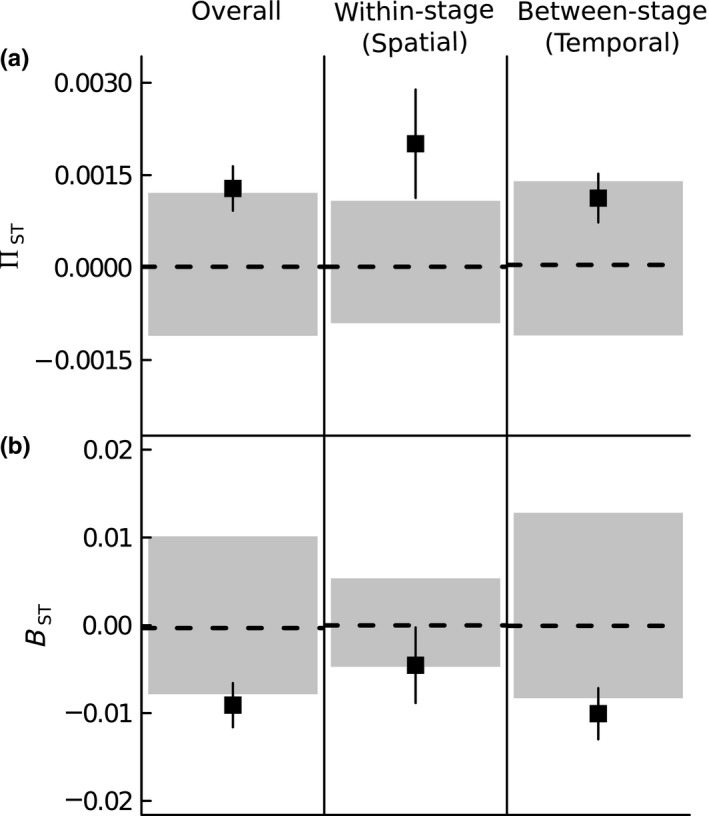
Phylogenetic turnover for all pairs of plots (combining spatial and temporal turnover, *n *= 351, left panel) dissected into spatial, i.e. within‐successional stage, (*n *= 62, middle panel) and temporal, i.e. between‐stage, (*n *= 289, right panel) turnover (black squares, mean ± 1 SE). Phylogenetic turnover was calculated for (a) presence/absence (Π_ST_) and (b) abundance data (B_ST_) and is based on the partitioning of the mean phylogenetic distance between distinct species, or between individuals of distinct species, into within‐ and between‐community components. Π_ST_, or B_ST_, > 0 indicate that the species, or individuals, co‐occurring within communities are phylogenetically more related to each other than to species from other communities (high turnover). B_ST_, or Π_ST_, < 0 indicate that the species, or individuals, co‐occurring within communities are phylogenetically less related to each other than to species from other communities (low turnover). The black‐dashed line and gray‐shaded area represent the mean and the 95% CI, respectively, from the 999 random communities. B_ST_ and Π_ST_ values outside the interval indicate nonrandom phylogenetic turnover

Levels of overall phylogenetic turnover were significantly different from those predicted, given the levels of species turnover (Figure 2). However, deviation from null expectations showed opposing patterns depending on whether phylogenetic turnover was estimated based on species presence/absence (Π_ST_) or abundance (B_ST_). Overall levels of presence/absence‐based turnover were higher than expected, whereas overall abundance‐based turnover was lower than expected. When overall phylogenetic turnover was dissected into turnover between pairs of plots belonging to the same successional stage (within‐stage spatial turnover) and turnover between pairs of plots at different successional stages (between‐stage temporal turnover), respectively, presence‐/absence‐based within‐stage turnover (Π_ST_) was higher than expected, indicating that species within plots were more closely related to each other than to species from different plots. Levels of presence‐/absence‐based between‐stage turnover (Π_ST_) did not differ from random expectations (Figure 2a). In contrast, between‐stage turnover was on average lower than predicted by chance, when based on abundance data (B_ST_).

### 3.3 Phylogenetic turnover within and between single successional stages

**Figure 3:**
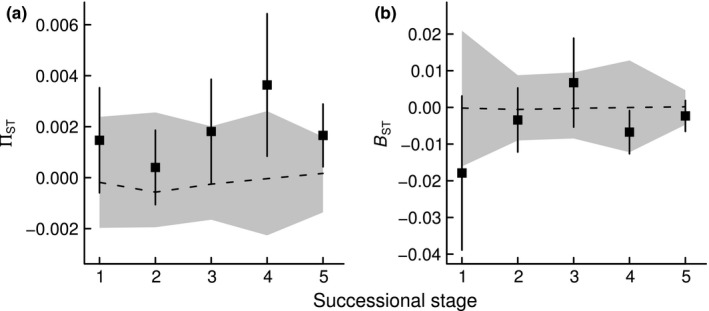
Spatial phylogenetic turnover between all pairs of communities within each of the five successional stages (black squares, mean ± 1 SE; Stage 1: *n *= 10, Stage 2: *n *= 6, Stage 3: *n *= 10, Stage 4: *n *= 15, Stage 5: *n *= 21), based on (a) presence/absence (Π_ST_) and (b) abundance data (B_ST_). B_ST_ or Π_ST_ values above (or below) the gray‐shaded area (i.e., the 95% CI for the B_ST_ or Π_ST_ values from the 999 random communities) indicate spatial phylogenetic clustering (or overdispersion)

Spatial phylogenetic turnover measures showed contrasting patterns of deviation from random expectations over the course of succession (Figure 3). Presence‐/absence‐based phylogenetic turnover (Π_ST_) did not significantly differ from zero within early and mid‐successional stages (stages 1, 2, and 3, Figure 3a). However, Π_ST_ values were higher than expected within the two latest successional stages (stages 4 and 5, Figure 3a). In contrast, abundance‐based spatial phylogenetic turnover (B_ST_) was lower than predicted by chance within the first successional stage but did not significantly differ from null expectations within the mid‐ and late‐successional stages (Figure 3b). Presence‐/absence‐based turnover (Π_ST_) between pairs of consecutive successional stages was higher than expected between the mid‐ and last successional stages (stages 3–4, stages 3–5, and stages 4–5, Fig. S3) but was never higher than levels of turnover within each of the stages 3, 4, and 5 (Figure 3a). Presence‐/absence‐based turnover (B_ST_) was lower than predicted between the early‐ and mid‐successional stages as well as between the first and the last stage (stages 1–2 and stages 1–5, Fig. S3), with values of B_ST_ that were lower than those estimated within stages (Figure 3b).

### 3.4 Covariation between phylogenetic turnover and environmental differentiation

**Figure 4:**
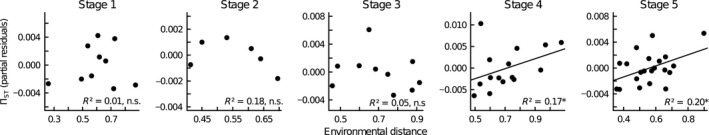
Relationship between presence/absence‐based phylogenetic turnover (Π_ST_) and environmental differences (with respect to topography, light, and soil characteristics) between communities, within each of the five successional stages. Π_ST_ values are given as partial residuals after accounting for spatial distance as a covariable. R² values are given. Significant relationships (based on randomization testing) are indicated by solid lines and are only detected in the two late‐successional stages. *p ≤ .05, n.s. not significant

There were no significant relationships of presence‐/absence‐based overall phylogenetic turnover and between‐stage phylogenetic turnover (Π_ST_), respectively, with environmental differences between plots (Fig. S4a,c). Instead, there was on average a significant positive association between within‐stage phylogenetic turnover (Π_ST_) and environmental distance (Fig. S4b), indicating an increase in phylogenetic turnover with increasing environmental differences (mainly related to soil moisture and light, see Table S6 and Fig. S7), between plots that belong to the same successional stage. When relationships between Π_ST_ and environmental distance were assessed within each of the five successional stages separately, significant increases in phylogenetic turnover with increasing environmental distance were only detected within the two last successional stages (stages 4 and 5, Figure 4). The significant positive associations between phylogenetic turnover and environmental differences between plots within the two latest successional stages were maintained after accounting for differences in sample size between the stages using resampling down to the minimum number of plots (*n *= 4) across stages (Stage 4: R² = 0.24*; Stage 5: R² = 0.19*). Abundance‐based phylogenetic turnover (B_ST_) was not associated with environmental distances, neither within nor between successional stages (results not shown).

### 3.5 Phylogenetic structure at different depths in the phylogeny

**Figure 5:**
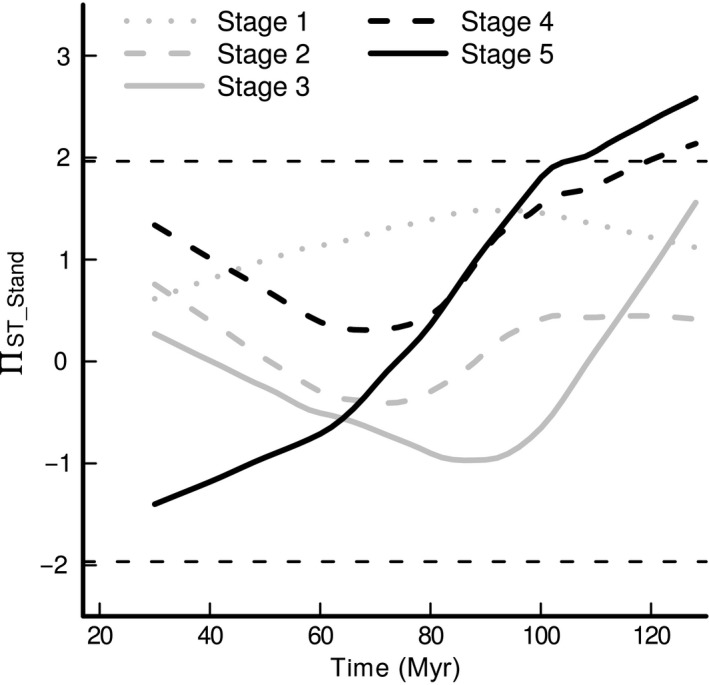
Phylogenetic turnover, based on presence/absence data (Π_ST_), at different phylogenetic depths, within the five successional stages. The lines represent, for each successional stage, fitted curves from local polynomial regression (loess, smoothing span = 0.66, polynomial degree = 1), of node age against the standardized effect size of phylogenetic turnover (Π_ST___Stand_). Π_ST___Stand_ values were calculated as the ratio between observed to expected values of Π_ST_: Π_ST___Stand_ =  (Π_ST___obs_ ‐Π_ST___exp_)/sd(Π_ST___exp_), where Π_ST___obs_ is the observed Π_ST_ value at a particular node, and Π_ST___exp_ and sd(Π_ST___exp_) are the mean and standard deviation of the expected Π_ST_ values from 999 partial phylogenetic tree randomizations among clades younger than that particular node. The two horizontal dashed lines indicate the 0.05 significance levels. Nonrandom and higher‐than‐expected turnover (spatial phylogenetic clustering) was only detected within the two late‐successional stages and at broad phylogenetic scales (from approximately 128–100 Myr)

Presence‐/absence‐based phylogenetic turnover (Π_ST_) within the early‐ and mid‐successional stages did not differ from random expectations throughout the phylogeny (Figure 5). Nonrandom and higher‐than‐expected phylogenetic turnover was only detected within the two latest successional stages (stages 4 and 5, Figure 5) and occurred close to the root of the phylogeny (>100 Myr), indicating phylogenetic clustering at a deep phylogenetic scale. Abundance‐based phylogenetic turnover (B_ST_) did not differ from random expectations at any level in the in phylogeny within any successional stage (results not shown). Clades that were overrepresented in, and contributed to the high turnover between, pairs of plots within the late‐successional stages diverged early in phylogeny (~100 Myr ago). Nodes that were significantly associated (i.e., had more taxa than expected by chance) with each of the plots are listed in Table S8. For instance, the plot pair with the highest level of phylogenetic turnover within the late‐successional stage 4 (plot IDs CSPs 5 and 11), (i) had significantly more taxa than expected within the families Ericaceae (*Rhododendron*,* Vaccinium*,* Lyonia*, and *Pieris*) and Theaceae (*Camellia*,* Schima*) (nodes 44 & 39) that diverged within the Ericales ~100 Myrs ago (Fig. S6) and (ii) was associated with dry and moist soil conditions, respectively (Fig. S7).

### 3.6 Phylogenetic signal in traits

All of the six traits considered showed significant phylogenetic signal, with values of Blomberg's *K*, Pagel's λ*,* and Abouheif/Moran's *I* significantly greater than expected from a null model of no phylogenetic signal (Table S7). This suggests that, in our study, phylogenetic relatedness reflects overall trait similarity.

## 4 DISCUSSION

The present study combines analysis of within‐ and between‐stage phylogenetic turnover during succession across phylogenetic scales, while accounting for between‐plot environmental differentiation, and demonstrates that, despite a lack of temporal phylogenetic turnover between stages, there was a shift from abundance‐based phylogenetic overdispersion in early succession toward presence‐/absence‐based phylogenetic clustering in late succession. Low between‐stage turnover that was not explained by environmental differences between stages suggests that (i) relatively constant environmental conditions and (ii) shifts in species abundances (toward higher evenness) that were counterbalanced by increasing relatedness toward late succession, resulted in an absence of net change in phylogenetic composition over time. Within the late‐successional stages, phylogenetic turnover was higher than expected, increased with environmental differentiation between sites and occurred at broad phylogenetic scales, indicating (i) deep phylogenetic conservatism of species’ abiotic niches, and (ii) that environmental filtering along an abiotic gradient becomes more important toward late succession.

### 4.1 Comparisons between spatial and temporal phylogenetic turnover: high turnover within and low turnover between successional stages

Within‐stage and between‐stage phylogenetic turnovers showed, on average, opposing levels of deviation from random, depending whether they were based on presence/absence or abundance data. While turnover between plots belonging to the same successional stage was higher than expected, relative to the levels of species turnover, when based on presence/absence data, phylogenetic turnover between plots at different successional stages was lower than expected when based on abundance data (Figure 2). Preceding studies (Fine & Kembel, 2011; Lozupone et al., 2007) have demonstrated that using both presence‐/absence‐ and abundance‐based metrics may reveal different patterns of phylogenetic structure for rare and abundant species and thus may help to distinguish species composition from dominance effects. The previous study by Norden et al. (2012) revealed that temporal changes in phylogenetic community structure during tropical rainforest succession were influenced by shifts in species’ abundance rather than species occurrence, whereas Letten et al. (2014) found low temporal phylogenetic turnover during heathland succession, because closely related, dominant species replaced each other over time. The previous study of Bruelheide et al. (2011), in the same system that was used in our study, demonstrated a lack of species turnover with only few species restricted to a particular successional stage, reminiscent of the concept of initial floristic composition, but that there were substantial shifts in species’ abundance toward a more even distribution of abundance in late‐successional communities. Therefore, in our study, the low levels of abundance‐based phylogenetic turnover, relative to the turnover of species between successional stages, reflect the fact that the temporal increase in evenness is counterbalanced by the increase in relatedness between the most dominant species toward late succession (Figures 1c and S2): the most dominant species within the early‐successional stages *(Loropetalum chinense*,* Quercus serrata*,* Rhododendron simsii*) are distantly related, whereas late‐successional communities were comprised of closely related species, belonging to the genera *Castanopsis*,* Rhododendron*,* Camellia,* and *Eurya*, respectively—resulting in an absence of a net change in phylogenetic diversity and composition over time. Further, low levels of temporal functional turnover during tropical forest succession were detected in an earlier study by Swenson et al. (2012), presumably due to relatively constant local environmental conditions through time. In our study, environmental differences between communities at different successional stages were similar to those between communities at the same stage (Fig. S4b,c), indicating that the lack of phylogenetic shifts likely reflects the constant abiotic conditions throughout succession.

In spite of the lack of temporal phylogenetic turnover between stages, we found a higher‐than‐expected presence‐/absence‐based phylogenetic turnover (Π_ST_) between plots that belong to the same successional stage, suggesting that there are filtering processes that have selected for different groups of closely related species. Our finding that the within‐stage phylogenetic turnover (Π_ST_) significantly increased with environmental distance (Fig. S4b) indicates that phylogenetic differentiation between communities belonging to the same successional stage was due to an underlying environmental gradient (mainly related to soil moisture and light; see Table S6), and that the higher‐than‐expected levels of spatial phylogenetic turnover reflect differential abiotic filtering selecting for closely related species within communities that belong to the same successional stage (see following section). The strong association between within‐stage phylogenetic turnover and environmental differences may also be a reflection of the fact that, in contrast to previous studies of community turnover in subtropical forest systems that have focussed on indirect abiotic descriptors such as elevation or habitat types (Legendre et al., 2009), we used a large set of environmental (edaphic, light, and topographic) descriptors. And it has been demonstrated recently that the quality of environmental data may influence conclusions about assembly processes (Chang, Zelený, Li, Chiu, & Hsieh, 2013).

### 4.2 Temporal changes in within‐stage turnover

We found that there was a shift from (abundance‐based) spatial phylogenetic overdispersion within the first successional stage toward (presence/absence‐based) spatial phylogenetic clustering within the two late‐successional stages (Figure 3). This contrasts with a number of previous studies of successional tropical and subtropical forests (Ding et al., 2012; Letcher, 2010; Norden et al., 2012; Whitfeld, et al., 2012) that found high levels of phylogenetic relatedness in young, disturbed forest communities, compared to older communities. Those studies concluded that disturbance in early succession acts as an abiotic filter and selects for closely related species but that competitive exclusion of closely related species becomes increasingly important towards late succession. Our finding that the most dominant species within plots were less related to each other than to species from different plots within the first successional stage may be explained in a number of different ways: First, phylogenetic overdispersion may reflect abiotic filtering if the traits conferring environmental tolerance are not phylogenetically conserved and distantly related species are filtered by the same environment (Cavender‐Bares et al., 2004). However, we detected significant phylogenetic signal in a set of six traits reflecting multiple axes of plant functional differentiation, and Eichenberg, Purschke, Ristok, Wessjohann, and Bruelheide (2015) found even stronger phylogenetic signal in the same study system when intraspecific trait variation was taken into account. This indicates that phylogenetic relatedness reflects ecological similarity between species and that abiotic filtering of convergent niche traits is unlikely to explain phylogenetic overdispersion in our study. Second, phylogenetic overdispersion may result from competitive exclusion of closely related species that share similar traits—a process that is expected to result in overdispersion at small phylogenetic scales. However, in our study, we did not detect phylogenetic overdispersion at shallow phylogenetic depth (Figure 5). Third, it has recently been demonstrated that early‐successional communities may be comprised of distantly related species in cases where (i) early‐successional pioneers are distributed all over the phylogeny (Letcher et al., 2015) and/or (ii) remnant species, which have persisted from former management, have a wide range of phylogenetically conserved traits that allow them to tolerate early‐successional environmental conditions (Bhaskar, Dawson, & Balvanera, 2014). Because in our study, (i) most species were present throughout succession, and (ii) remnant species were represented by only a few individuals (e.g., *Nyssa sinensis*,* Castanea henryi*,* Cyclobalanopsis glauca, and Castanopsis fargesii*; see Figure 1c & Bruelheide et al., 2011) and hence did not substantially contribute to abundance‐based phylogenetic structure, the abundance‐based phylogenetic overdispersion in early succession is unlikely to reflect the presence of pioneer or remnant species. Finally, phylogenetic overdispersion may reflect successful dispersal of species that have different dispersal strategies (Du, Mi, & Ma, 2012), provided that dispersal traits are phylogenetically conserved (Baeten, Davies, Verheyen, Van Calster, & Vellend, 2015). In our study, the most abundant species within the first successional stage (e.g., *Loropetalum chinense, Quercus serrata, and Rhododendron simsii*) were both, distantly related (Figure 1c) and dispersed by different dispersal modes (animal‐dispersed acorns, ballistic‐ and wind‐dispersed seeds for *Quercus*,* Loropetalum,* and *Rhododendron*, respectively), suggesting that the abundance‐based phylogenetic overdispersion in early succession likely reflects the coexistence of a wide range of different dispersal strategies (Levin & Muller‐Landau, 2000; Purschke et al., 2014).

Within the two late‐successional stages, presence‐/absence‐based phylogenetic turnover was higher than expected relative to the levels of species turnover, indicating deterministic filtering that selects for distinct sets of closely related species in the different plots. There are a few studies that found increasing functional similarity in (sub)tropical forest communities over time (Buzzard et al., 2016; Uriarte et al., 2010), concluding that the relative importance of abiotic filtering increases with forest age. Further, the previous studies by Hardy et al. (2012) and Fine and Kembel (2011), focussing on phylogenetic turnover between tree communities along environmental gradients, pointed out that, if environmental niches are evolutionarily conserved, abiotic filtering is predicted to result a in strong covariation between phylogenetic turnover and environmental differentiation between plots (Cadotte & Tucker, 2017). Therefore, our finding that phylogenetic turnover within late‐successional stages was higher than expected and explained by environmental differentiation [mainly related to soil and light conditions (Table S6), and independent of spatial distance] between plots (Figure 4), is consistent with phylogenetic niche conservatism and indicates that the relative importance of environmental filtering along an environmental gradient increased during the course of succession. The high phylogenetic turnover within the late‐successional stages, together with the lack of temporal between‐stage phylogenetic turnover, further suggests that phylogenetic clustering in late succession reflects the local colonization of species that (i) are closely related to residents (Li et al., 2015) and (ii) were already present in the early‐successional species pool, indicating that species sorting into their preferred habitat takes time to develop. Spatial phylogenetic clustering in late succession was only detected close to the root of the phylogenetic tree (Figure 5). Previous studies of community turnover across phylogenetic scales (Cavender‐Bares & Reich, 2012; Parmentier & Hardy, 2009) found that phylogenetic turnover increased both with phylogenetic depth as well as with environmental differentiation between sites, and concluded that ancient diversification events, together with niche conservatism, still show an imprint on the assembly of current plant communities. The fact that, in our study, spatial phylogenetic clustering (Π_ST_) within late‐successional communities was only detected at large phylogenetic scales (i.e., between taxa that diverged >100 Myrs years ago), together with the finding that phylogenetic turnover was explained by abiotic differences (related to soil and light conditions) between plots is consistent with deep phylogenetic signal in species’ soil moisture and light niche. Clades that contributed to the high phylogenetic turnover within the late‐successional stage diverged early in phylogeny and were associated with one or the other end of the environmental gradient (Table S8, Fig. S7), indicating environmental niche differentiation between species that diverged early in phylogeny.

Alternatively, phylogenetic clustering in late succession can also result from hierarchical competition if early‐successional pioneers are replaced by competitively superior closely related species in late succession (Kunstler et al., 2012; Letten et al., 2014). However, most early‐successional species in our study were still present in late succession. Further, hierarchical competition is predicted to result in phylogenetic clustering that is unrelated to environmental differentiation between plots (Bartlett et al., 2016), which was not the case in our study. This suggests that competition hierarchies are unlikely to explain the phylogenetic clustering in our study. Our finding that nonrandom phylogenetic structure within the two latest successional stages was only detected based on presence/absence data (Figure 3a), is likely to reflect the high number of rare species found in late compared to early succession (Figure 1c, see also Bruelheide et al., 2011), and in such situations presence/absence metrics (such as Π_ST_), giving high weight to rare species, will provide greater testing power to detect significant community phylogenetic structure than metrics based on abundance (Helmus et al., 2007; Vellend et al., 2011).

In conclusion, the integrated analysis of the spatial and temporal components of phylogenetic relatedness during succession, across phylogenetic and environmental scales, allowed to test competing hypothesis about the temporal dynamics of community processes after disturbance. Our results do not support a model that predicts a progression toward decreasing phylogenetic relatedness over time. Instead, our findings support a deterministic model of community assembly where the phylogenetic composition is constrained though time but different assembly processes act at different ends of the successional gradient: Colonization of species that differ in their dispersal strategies likely plays an important role in early succession, whereas, despite the lack of phylogenetic shifts between stages, environmental filtering of niche traits that are conserved deep in phylogeny becomes increasingly important toward late succession. Such insights into the temporal dynamics of postdisturbance community assembly processes were not apparent from previous analyses that focused either on single (spatial or temporal) phylogenetic turnover components or single phylogenetic scales.

## CONFLICT OF INTEREST

None declared.

## AUTHOR CONTRIBUTIONS

H.B. established the BEF‐China experimental platform. O.P. developed the main idea for this manuscript with contributions from W.D., S.G.M., and H.B. O.P. analyzed the data and interpreted the results with input from all co‐authors. S.G.M. generated the phylogenetic tree with contributions from W.D. O.P. wrote the first draft of the manuscript with all other authors substantially contributing to revisions.
