# Supplementary material for: Phylogenetic turnover during subtropical forest succession across environmental and phylogenetic scales
Source: Ecol Evol. 2017 Nov 15;7(24):11079–91. doi: 10.1002/ece3.3564 (PMC5743486; doi:10.1002/ece3.3564)
Supplement: Supplementary file 1 [file ECE3-7-11079-s001.doc]

## Supporting Information

Article title: **Phylogenetic turnover during subtropical forest succession across environmental and phylogenetic scales**

Authors: Oliver Purschke, Stefan G. Michalski, Helge Bruelheide, Walter Durka.

The following Supporting Information is available for this article:

**Fig. S1** Rarefaction curves (estimated species richness of each plot).

**Fig. S2** Shannon evenness within each of the five successional stages.

**Fig. S3** Temporal phylogenetic turnover between single successional stages.

**Fig. S4** Relationship between phylogenetic turnover (Π_ST_; (a) overall, (b) within-stage, (c) between-stage) and environmental differentiation.

**Fig. S5** Presence/absence-based phylogenetic turnover (Π_ST_) at different phylogenetic depths.

**Fig. S6** Illustration of results from the nodesig analysis.

**Fig. S7** PCA of environmental variables.

**Table S1** Correlations between rarefied and non-rarefied phylogenetic diversity measures.

**Table S2** Pearson correlations between environmental descriptors and successional stage.

**Table S3** Species pool including the 438 woody species of the Gutianshan National Nature Reserve.

**Table S4** Sequence information for the woody plant species in the Gutianshan National Nature Reserve.

**Table S5** Age constraints for nodes used to create the ultrametric tree.

**Table S6** PCA scores of environmental variables.

**Table S7** Phylogenetic signal (Blomberg's *K*, Pagel's *λ* and Abouheif/Moran's *I*) in traits.

**Table S8** Nodes that are significantly associated with the single plots (CSPs).

**Methods S1** Phylogenetic inference.

**Methods S2** Maximum likelihood phylogenetic tree for the woody plant species in Gutianshan.

**Methods S3** Dated, ultrametric, phylogenetic tree for the woody plant species in Gutianshan.

**Methods S4** Simulation procedure to assess the relationship between phylogenetic turnover (Π_ST_ or B_ST_) and the number of plots.

**Methods S5**  Testing for non-random structure in species frequencies and abundances.

**Fig. S1** Rarefaction curves of the 27 woody plant communities (CSPs), giving the estimated number of species for any number of individuals. The five successional stages are indicated by different line colors. The vertical line depicts the minimal number of individuals (n=175) sampled in a plot. The intersection between the rarefaction curves and the vertical line corresponds to the estimated number of species if only 175 individuals per plot were sampled.

**Fig. S2** Shannon evenness within each of the five successional stages (black squares, mean ± 1 SE). R²-value is given. The solid line indicates the significant relationship between evenness and successional stage. ** P ≤ 0.01.

**Fig S3** Phylogenetic turnover between successional stages (black squares, mean ± 1 SE), calculated for (a) presence/absence (Π_ST_) and (b) abundance data (B_ST_). The black dashed line and grey-shaded area represent the mean and the 95% CI, respectively, from the 999 random communities. B_ST_ and Π_ST_ values above the interval indicate higher than expected temporal phylogenetic turnover. B_ST_ and Π_ST_ values below the interval indicate lower than expected temporal phylogenetic turnover.

**Fig S4** Relationships between presence/absence-based phylogenetic turnover and environmental differences (with respect to topography, light and soil characteristics) between communities for (a) all pairs of plots (combining spatial and temporal turnover, n=351), (b) pairs of plots of the same successional stage (spatial turnover, n=62) and (c) pairs of plots belonging to different successional stages (temporal turnover, n=289). Π_ST_ values are given as partial residuals after accounting for spatial distance as a covariable. R² values are given. The significant relationship (based on randomization testing) between spatial phylogenetic turnover and environmental distance is indicated by the solid red line. * P ≤ 0.01, . P ≤ 0.1, n.s. not significant.

**Fig S5** Phylogenetic turnover, based on presence/absence data (Π_ST_), at different phylogenetic depths, within the five successional stages. Standardized Π_ST_ values (ΠST_Stand) are given, calculated as the ratio between observed to expected values of Π_ST_: ΠST_Stand=(ΠST_obs -ΠST_exp)/sd(ΠST_exp), where ΠST_obs is the observed Π_ST_ value at a particular node, and ΠST_exp and sd(ΠST_exp) are the mean and standard deviation of the expected Π_ST_ values from 999 partial phylogenetic tree randomizations among clades younger than that particular node. The dashed lines indicate the 0.05 significance levels. Non-random and higher-than-expected turnover (spatial phylogenetic clustering) was only detected within the two late successional stages and at broad phylogenetic scales (from approximately 128 to 100 Myr).

**Fig. S6** Illustration of results from the nodesig analysis. Highlighted are clades (shaded areas) that had significantly more taxa than expected in plot pairs with the highest levels of phylogenetic turnover at the two latest successional stages (blue: plot pair CSP 5 & 11 at stage 4; red: CSPs 4 & 12 at stage 5, see also Fig. 1c). For instance, node N39 and N44, respectively, i) were significantly associated with the plots CSPs 5 and 11 (the plot pair that that had the highest phylogenetic turnover in stage 4, see also Table S8), and ii) correspond to the families Theaceae (*Camellia, Schima*) and [Ericaceae](https://en.wikipedia.org/wiki/Ericaceae) (*Rhododendron, Vaccinium, Lyonia, Pieris*), that diverged early in phylogeny ~100 Myrs ago within the Ericales at node N22 (red vertical line). See Table S8 for a complete list of nodes that were significantly associated with each of the plots.

**
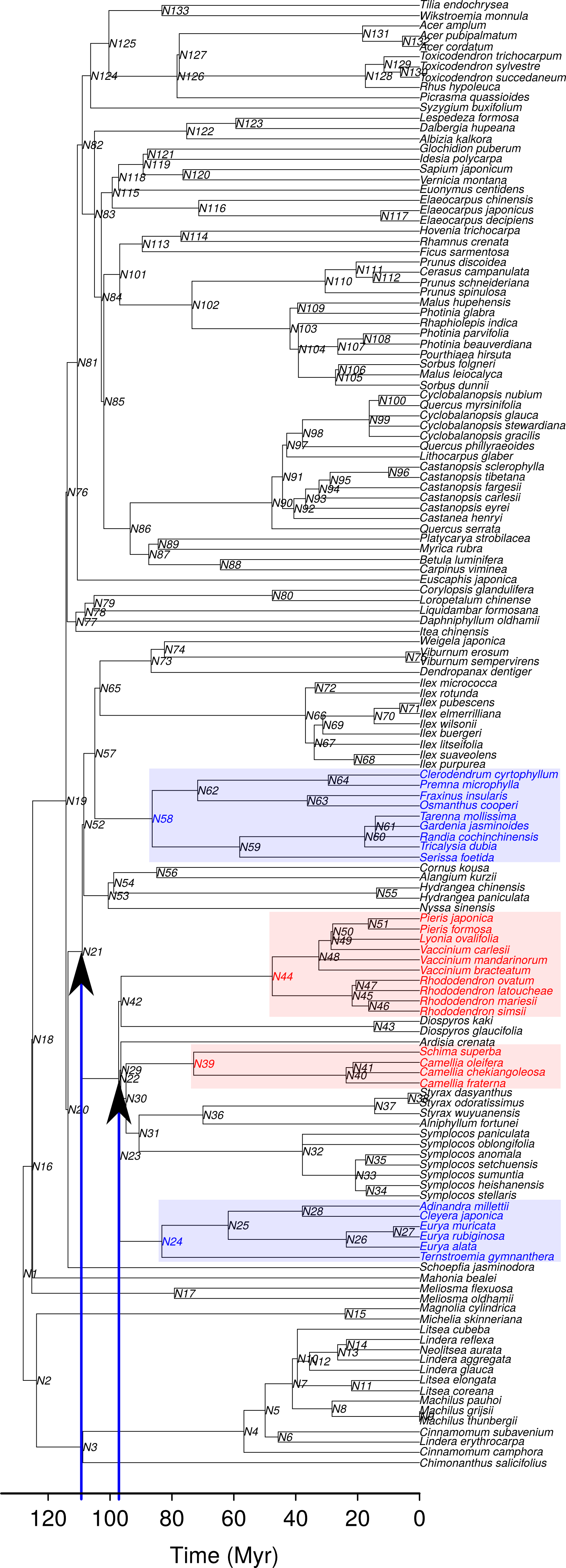
**

**Fig. S7.** PCA biplot illustrating the association between the 11 environmental variables and the 27 plots (CSPs). See Table S6 for variable loadings and Table S2 for Pearson correlations.

**Table S1** Correlations between non-rarefied and rarefied phylogenetic metrics. Estimates of phylogenetic diversity and turnover were recalculated (100 times) for rarefied communities containing 175 individuals each (the minimum number of individuals recorded in a plot). Rarefied and non-rarefied estimates for all of the metrics were strongly (P < 0.05) correlated.

|  | **Correlation** | |
| --- | --- | --- |
| **Metric** | **Mean** | **SD** |
| **Δ^P^_w_** | 0.821 | 0.036 |
| **Δ^*P^_w_** | 0.970 | 0.006 |
| **Π_ST_** | 0.632 | 0.049 |
| **B_ST_** | 0.967 | 0.004 |

**Table S2** Pearson correlations between successional stage and the 11 abiotic environmental descriptors and successional stage. Significant correlations (P < 0.05) are highlighted in bold.

|  | **Elevation** | **Aspect_E** | **Aspect_N** | **Slope** | **Light (PAR)** | **Red/far-red** | **Soil moisture** | **pH** | **Soil C/N** | **N mineral** | **P total** |
| --- | --- | --- | --- | --- | --- | --- | --- | --- | --- | --- | --- |
| **Stage** | 0.29 | -0.26 | -0.26 | 0.11 | **-0.41** | **0.59** | 0.29 | -0.36 | 0.1 | **0.59** | 0.07 |
| **Elevation** |  | -0.29 | 0.03 | -0.11 | -0.28 | 0.21 | **0.51** | **-0.43** | -0.06 | **0.47** | 0.29 |
| **Aspect_E** |  |  | 0.24 | 0.23 | **0.39** | -0.31 | -0.21 | 0.11 | 0.1 | -0.18 | 0.03 |
| **Aspect_N** |  |  |  | **0.4** | 0.21 | -0.21 | 0.15 | -0.26 | 0.13 | -0.18 | 0.28 |
| **Slope** |  |  |  |  | 0.04 | -0.06 | 0.33 | -0.15 | 0.05 | 0.14 | **0.48** |
| **Light (PAR)** |  |  |  |  |  | **-0.82** | 0.14 | 0.09 | -0.27 | -0.25 | 0.12 |
| **Red/far-red** |  |  |  |  |  |  | -0.13 | -0.27 | **0.38** | 0.3 | -0.18 |
| **Soil moisture** |  |  |  |  |  |  |  | -0.34 | **-0.43** | **0.5** | **0.83** |
| **pH** |  |  |  |  |  |  |  |  | -0.24 | **-0.4** | -0.15 |
| **Soil C/N** |  |  |  |  |  |  |  |  |  | -0.13 | **-0.58** |
| **N mineral** |  |  |  |  |  |  |  |  |  |  | 0.31 |

**Table S4** Sequence information for the woody plant species in the Gutianshan National Nature Reserve.

|  |  | **GeneBank accession number** | |  |  |
| --- | --- | --- | --- | --- | --- |
| **Species** | **Species substitute or synonym** | ***mat*K** | ***rbc*L** | **5.8s+ITS** |  |
| *Abelia_chinensis* |  | AY310461 | HQ680737 | FJ745388 |  |
| *Abutilon_theophrasti* |  | HM850990 | HM849734 | DQ006017 |  |
| *Acanthopanax_trifoliatus* |  | U58603 | U50239 |  |  |
| *Acer_amplum* | *Acer campestre* | JN894032 | DQ978399 | DQ238431 |  |
| *Acer_buergerianum* |  |  | DQ978396 | U89908 |  |
| *Acer_cordatum* | added manually to ML tree |  |  |  |  |
| *Acer_davidii* |  | JF952989 | DQ978406 | JF975773 |  |
| *Acer_elegantulum* |  | HQ427339 | HQ427191 |  |  |
| *Acer_mono* |  |  | DQ978416 | AY605447 |  |
| *Acer_olivaceum* |  | HQ427338 |  |  |  |
| *Acer_pubipalmatum* | added manually to ML tree |  |  |  |  |
| *Acer_tataricum* |  |  | DQ978436 | AY605363 |  |
| *Acer_wilsonii* |  | HQ427337 | HQ427189 | HM352665 |  |
| *Actinidia_callosa* |  | AF322620 | AJ549061 | AF323829 |  |
| *Actinidia_chinensis* |  | U61324 | L01882 |  |  |
| *Actinidia_hemsleyana* |  | AF322608 | AJ549036 | AF323802 |  |
| *Actinidia_lanceolata* |  |  | AJ549072 |  |  |
| *Actinidia_melanandra* |  | AF322600 |  | AF443211 |  |
| *Adina_rubella* |  |  | AJ346965 | AJ346856 |  |
| *Adinandra_millettii* |  | AF380069 | HQ427223 | AY626848 |  |
| *Aesculus_chinensis* |  | EU687709 |  | JF421459 |  |
| *Ailanthus_altissima* |  | EF489111 | HM849750 | JF755934 |  |
| *Akebia_quinata* |  | AF542587 | L12627 | GQ339575 |  |
| *Akebia_trifoliata* |  | GQ434168 | AF335305 | AY029788 |  |
| *Alangium_kurzii* |  | FJ644650 | DQ340449 | FJ610018 |  |
| *Alangium_platanifolium* |  | FJ644640 | JF308649 | FJ610006 |  |
| *Albizia_julibrissin* |  | AY386855 | GU135262 | FJ572041 |  |
| *Albizia_kalkora* |  | HQ427295 | HQ427141 | JF708202 |  |
| *Alniphyllum_fortunei* |  | HQ427279 | AF396149 | AF396437 |  |
| *Amelanchier_asiatica* |  |  |  | JQ392362 |  |
| *Antidesma_japonicum* | *Antidesma venosum* | HQ415372 | JF265291 |  |  |
| *Aphananthe_aspera* |  | AF345320 | AF500339 |  |  |
| *Aralia_chinensis* |  | HQ427393 | HQ427250 | U63181 |  |
| *Aralia_dasyphylla* |  |  |  | DQ007355 |  |
| *Aralia_echinocaulis* |  |  |  | AF273525 |  |
| *Ardisia_brevicaulis* |  |  |  | FJ482141 |  |
| *Ardisia_crenata* |  | HQ427412 | L12599 | JN645186 |  |
| *Ardisia_crispa* |  |  |  | FJ482139 |  |
| *Ardisia_hanceana* |  |  |  | JN645190 |  |
| *Ardisia_japonica* |  | JF416274 | GQ436756 | JN645201 |  |
| *Berberis_soulieana* | *Berberis fortunei* |  | FJ449857 | FJ980428 |  |
| *Berchemia_huana* | *Berchemia zeyheri* | JF270656 | JF265303 |  |  |
| *Betula_luminifera* |  | FJ011821 |  | AY761116 |  |
| *Bischofia_polycarpa* | *Bischofia javanica* | GU135116 | AY663571 |  |  |
| *Broussonetia_papyrifera* |  | AF345326 | JF317478 | HM623778 |  |
| *Buddleja_lindleyana* | *Buddleja davidii* | HQ384530 | AJ001757 |  |  |
| *Buxus_sinica* | *Buxus sempervirens* | AF543728 | HM849831 | EF123195 |  |
| *Caesalpinia_decapetala* |  | HM049555 |  | JF708207 |  |
| *Callicarpa_bodinieri* |  | HQ427330 | HQ427182 |  |  |
| *Callicarpa_giraldii* |  | HQ427332 | HQ427184 | FJ593347 |  |
| *Callicarpa_japonica* |  | FM163257 |  | FM163230 |  |
| *Callicarpa_rubella* |  | HQ427329 | HQ427181 | FM163232 |  |
| *Camellia_brevistyla* |  |  |  | HM061465 |  |
| *Camellia_chekiangoleosa* |  | HQ427374 | HQ427229 | EU579685 |  |
| *Camellia_cuspidata* |  | HQ427370 | HQ427225 | EU579693 |  |
| *Camellia_fraterna* |  |  | HQ427224 | EU579705 |  |
| *Camellia_oleifera* |  |  | GQ436647 | HM061454 |  |
| *Camellia_sinensis* |  | AJ429305 | AF380037 | HM061514 |  |
| *Camptotheca_acuminata* |  | JF953409 | L11211 | JF976064 |  |
| *Campylotropis_macrocarpa* |  | AY386870 | EU717277 | GU572164 |  |
| *Caragana_sinica* |  | HM049541 | FJ537233 | FJ537284 |  |
| *Carpinus_londoniana* |  | AY211990 |  | AF432040 |  |
| *Carpinus_viminea* |  | AY212000 | HQ427161 | AF432058 |  |
| *Castanea_henryi* |  | EF057123 |  |  |  |
| *Castanea_mollissima* |  | EF057124 |  |  |  |
| *Castanea_seguinii* |  | AY263920 | AY263937 |  |  |
| *Castanopsis_carlesii* |  | AY040496 | HQ427175 | AY040372 |  |
| *Castanopsis_eyrei* |  | EF057125 | HQ427167 | EF057109 |  |
| *Castanopsis_fargesii* |  | EF057133 | HQ427173 | AY040383 |  |
| *Castanopsis_sclerophylla* |  | EF057137 |  | EF057106 |  |
| *Castanopsis_tibetana* |  | AY263921 | AY147096 |  |  |
| *Celastrus_aculeatus* |  |  |  | JQ424095 |  |
| *Celastrus_angulatus* |  | EU328938 |  | JQ424098 |  |
| *Celastrus_gemmatus* |  |  |  | JQ424102 |  |
| *Celastrus_oblanceifolius* |  |  |  | JQ424119 |  |
| *Celastrus_rosthornianus* |  | EU328940 |  | JQ424130 |  |
| *Celastrus_stylosus* |  |  |  | JQ424136 |  |
| *Celtis_biondii* |  | KF569895 | KF569888 | |  |
| *Celtis_tetrandra* |  |  | JF317479 |  |  |
| *Cephalotaxus_fortunei* |  | AF228109 | AY450863 |  |  |
| *Cephalotaxus_sinensis* |  | AF228110 | EF660728 |  |  |
| *Cerasus_campanulata* | syn. *Prunus campanulata* |  | AF411501 | AF318658 |  |
| *Chimonanthus_salicifolius* |  | HQ427325 | HQ427177 | AY786102 |  |
| *Choerospondias_axillaris* |  | HQ427341 | HQ427193 | GQ434625 |  |
| *Cinnamomum_camphora* |  | AJ247154 | L12641 | AY878325 |  |
| *Cinnamomum_chekiangense* |  | HQ427409 | HQ427267 |  |  |
| *Cinnamomum_subavenium* |  | HQ427408 | HQ427266 | GU598529 |  |
| *Cladrastis_wilsonii* | *Cladrastis sikokiana* |  | U74232 | JQ676968 |  |
| *Clerodendrum_bungei* |  |  |  | U77744 |  |
| *Clerodendrum_cyrtophyllum* |  | HQ427333 | HQ427185 | JF755940 |  |
| *Clerodendrum_trichotomum* |  | AF477760 | HQ427186 | U77771 |  |
| *Clethra_barbinervis* |  | AB697681 | AF421089 | AY190573 |  |
| *Cleyera_japonica* |  | HQ427371 | EU980811 | AF456257 |  |
| *Coptosapelta_diffusa* |  |  | EU145453 | DQ358882 |  |
| *Cornus_controversa* |  | U96893 | AF190433 | AY530918 |  |
| *Cornus_kousa* |  | DQ341345 | L14395 | DQ340555 |  |
| *Corylopsis_glandulifera* | syn. *Corylopsis hypoglauca* | HQ427314 | HQ427165 | EF456719 |  |
| *Corylopsis_sinensis* |  | AF013038 | AB237032 | EF456711 |  |
| *Crataegus cuneata* | *Crataegus monogyna* | JN893932 | JN890652 |  |  |
| *Cryptomeria_fortunei* |  | AB030117 |  |  |  |
| *Cunninghamia_lanceolata* |  | AB030125 | AY140260 |  |  |
| *Cyclobalanopsis_glauca* | syn. *Quercus glauca* | AB060062 | AB060571 | AY040458 |  |
| *Cyclobalanopsis_gracilis* | syn. *Quercus ciliaris* | HQ427318 | HQ427169 |  |  |
| *Cyclobalanopsis_nubium* | syn. *Quercus sessilifolia* | AB060068 | AB060577 |  |  |
| *Cyclobalanopsis_stewardiana* |  | KF569896 | KF569889 |  |  |
| *Cyclocarya_paliurus* |  | AY147098 | AY147094 | AF303817 |  |
| *Dalbergia_hupeana* |  | HQ427296 | U74236 | GU217673 |  |
| *Daphne_genkwa* | *Daphne laureola* | JN894952 | HM849946 | GQ167533 |  |
| *Daphniphyllum_macropodum* |  |  | AM183400 |  |  |
| *Daphniphyllum_oldhamii* |  | HQ427311 | HQ427162 | JN040993 |  |
| *Dendropanax_dentiger* |  | HQ427394 | HQ427251 | GU054694 |  |
| *Deutzia_glauca* | *Deutzia setchuenensis* | JF308687 | JF308658 |  |  |
| *Diospyros_glaucifolia* |  | HQ427382 | EU980694 | FJ624405 |  |
| *Diospyros kaki* |  | GQ434247 | EU980698 | FJ624403 |  |
| *Diospyros_morrisiana* |  | HQ427383 | HQ427240 |  |  |
| *Diospyros_oleifera* |  | AB174997 |  | AB175016 |  |
| *Diospyros_rhombifolia* |  | AB174999 | EU980741 | AB175018 |  |
| *Distylium_myricoides* |  | GU576683 | AM183408 | GU576648 |  |
| *Edgeworthia_chrysantha* |  |  | AJ297920 | AJ744932 |  |
| *Ehretia_thyrsiflora* |  |  | EU599831 |  |  |
| *Elaeagnus_glabra* |  |  |  | JQ062502 |  |
| *Elaeagnus_multiflora* |  |  |  | JQ062478 |  |
| *Elaeagnus_pungens* |  | GU135102 | GU135269 | JQ062488 |  |
| *Elaeagnus_umbellata* |  | AY257529 | HM849968 | JQ062486 |  |
| *Elaeocarpus_chinensis* |  |  | HQ427153 |  |  |
| *Elaeocarpus_decipiens* |  | HQ415261 | HQ415077 |  |  |
| *Elaeocarpus_japonicus* |  | HQ415264 | HQ415080 |  |  |
| *Eleutherococcus_gracilistylus* |  |  | GQ436710 | FJ980422 |  |
| *Emmenopterys_henryi* |  | FJ905360 | Y18715 | FJ984985 |  |
| *Euchresta_japonica* |  |  | AB127040 |  |  |
| *Euodia_faugeaii* | *Euodia hupehensis* | EF489105 | FN552679 |  |  |
| *Euonymus_alatus* |  | EU328950 |  | EU328755 |  |
| *Euonymus_carnosus* |  | HQ427389 | HQ427246 |  |  |
| *Euonymus_centidens* |  | HQ427390 | HQ427247 |  |  |
| *Euonymus_fortunei* |  | HQ393828 | HM755927 | HQ393699 |  |
| *Euonymus_myrianthus* |  | HQ427388 | HQ427245 | HQ393721 |  |
| *Euonymus_oblongifolius* | syn. *Euonymus nitidus* | HQ393835 | HQ427248 | JQ424144 |  |
| *Euonymus_oxyphyllus* |  | HQ393836 |  | HQ393704 |  |
| *Eurya_alata* |  |  |  | AF456259 |  |
| *Eurya_hebeclados* |  |  |  | AY626865 |  |
| *Eurya_loquaiana* |  | HQ427372 | HQ427227 | AY626870 |  |
| *Eurya_muricata* |  | HQ427373 | HQ427228 | AY626872 |  |
| *Eurya_nitida* |  |  |  | AY096026 |  |
| *Eurya_rubiginosa* |  | HQ427368 | HQ427222 | AY626877 |  |
| *Euscaphis_japonica* |  | DQ663628 | DQ307099 |  |  |
| *Fagus_engleriana* |  | AY042391 | JF941501 | AY232907 |  |
| *Fagus_longipetiolata* |  | AY042402 | JF941508 | AY232955 |  |
| *Fagus_lucida* |  | EF057139 | JF941510 | AY232963 |  |
| *Ficus_erecta* |  | HQ427366 | HQ427220 | HQ890729 |  |
| *Ficus_heteromorpha* |  |  | JF941536 |  |  |
| *Ficus_pandurata* |  | HQ415327 | HQ415153 |  |  |
| *Ficus_pumila* |  | HM851109 | AF500352 | AY063580 |  |
| *Ficus_sarmentosa* |  |  |  | AB485901 |  |
| *Firmiana_platanifolia* |  |  | AY328192 | AF460185 |  |
| *Fontanesia_fortunei* | syn. *Fontanesia phillyreoides* |  |  | AF534815 |  |
| *Forsythia_viridissima* |  | FJ263957 |  | AF534810 |  |
| *Fraxinus_chinensis* |  | HM171509 | DQ673301 | HQ705225 |  |
| *Fraxinus_insularis* |  | HQ427335 | HQ427187 |  |  |
| *Gardenia_jasminoides* |  | HQ427344 | GQ436564 | GQ434646 |  |
| *Gardneria_multiflora* |  |  |  | JF937929 |  |
| *Gleditsia_sinensis* |  | AM086835 |  | AF510019 |  |
| *Glochidion_puberum* |  | HQ427285 | AY663586 | AY936659 |  |
| *Gymnocladus_chinensis* |  |  |  | AF510033 |  |
| *Hamamelis_mollis* |  | AF128827 | L01922 | GU576659 |  |
| *Helwingia_japonica* |  | AJ430195 | L11226 | AF200593 |  |
| *Hibiscus_syriacus* |  | AF345329 | AY328174 | AF460188 |  |
| *Holboellia_coriacea* | *Holboellia grandiflora* | FJ626513 | AF398181 | AY029779 |  |
| *Hovenia_dulcis* |  |  |  | DQ146607 |  |
| *Hovenia_trichocarpa* |  | JF317429 | JF317489 | DQ146608 |  |
| *Hydrangea_angustipetala* |  | GU217336 |  |  |  |
| *Hydrangea_anomala* |  | GU369710 | AF323202 | JF976651 |  |
| *Hydrangea_chinensis* |  | KF569897 | KF569890 | AB377211 |  |
| *Hydrangea_paniculata* |  | HQ427310 | AB236036 |  |  |
| *Hydrangea_strigosa* | syn. *Hydrangea aspera* | AJ429277 | JF941958 | JF976653 |  |
| *Idesia_polycarpa* |  | FJ670040 | AF206781 | AJ006441 |  |
| *Ilex_buergeri* |  |  | FJ394593 | FJ394663 |  |
| *Ilex_cornuta* |  | GQ997309 | FJ394601 | EU647650 |  |
| *Ilex_elmerrilliana* |  |  | HQ427132 |  |  |
| *Ilex_ficoidea* |  | HQ427288 | HQ427133 | FJ394682 |  |
| *Ilex_latifolia* |  | HQ427289 | X98731 | DQ200798 |  |
| *Ilex_litseifolia* |  | KF569898 |  |  |  |
| *Ilex_macrocarpa* |  |  | AJ4927271 | AJ492689 |  |
| *Ilex_micrococca* |  | HQ427290 | X98721 | JF976691 |  |
| *Ilex_pubescens* |  | HQ427291 | AJ492722 | AJ492686 |  |
| *Ilex_purpurea* |  | HQ427292 | AJ492710 | FJ394708 |  |
| *Ilex_rotunda* |  | HQ415255 | X98720 | FJ394710 |  |
| *Ilex_suaveolens* |  | HQ427293 | HQ427139 |  |  |
| *Ilex_triflora* |  |  | AJ4927131 | AJ492675 |  |
| *Ilex_tsoi* |  |  | FJ394645 | FJ394718 |  |
| *Ilex_wilsonii* |  | HQ427294 | FJ394649 | FJ394722 |  |
| *Illicium_lanceolatum* |  | HQ427283 | HQ427126 | JQ180205 |  |
| *Indigofera_decora* |  |  |  | AF534797 |  |
| *Itea_chinensis* |  | HQ415356 | HQ415186 |  |  |
| *Jasminum_sinense* | *Jasminum nudiflorum* | AF531779 |  | AF361301 |  |
| *Juglans_cathayensis* |  | AF118028 |  |  |  |
| *Juniperus_chinensis* |  | HM024014 | HM024292 |  |  |
| *Juniperus_formosana* |  | HM024028 | HM024306 |  |  |
| *Kerria_japonica* |  | AB073686 | AF132893 |  |  |
| *Koelreuteria_bipinnata* |  |  | DQ978447 |  |  |
| *Lasianthus_japonicus* |  | HQ427345 | HQ427196 |  |  |
| *Lespedeza_buergeri* |  |  |  | JN402408 |  |
| *Lespedeza_cyrtobotrya* |  |  |  | JN402422 |  |
| *Lespedeza_dunnii* |  |  |  | JN402431 |  |
| *Lespedeza_floribunda* |  | HM049538 | GQ436353 | JN402438 |  |
| *Lespedeza_formosa* | syn. *Lespedeza thunbergii* |  | HQ427143 | JN402486 |  |
| *Ligustrum_lucidum* |  | EU669873 | GQ436542 | JF976848 |  |
| *Ligustrum_sinense* |  | JF830514 | JF830433 | JF830366 |  |
| *Lindera_aggregata* |  | AB442057 | HM019473 | AB470487 |  |
| *Lindera_erythrocarpa* |  | AB259065 |  | HQ697215 |  |
| *Lindera_glauca* |  | AB442056 | HM019478 | AB500615 |  |
| *Lindera_megaphylla* |  | AF244404 |  | AY265406 |  |
| *Lindera_reflexa* |  | AF244401 | HQ427264 | AY265407 |  |
| *Liquidambar_acalycina* |  | AF015649 | DQ352380 | GU576668 |  |
| *Liquidambar_formosana* |  | AF133221 | AJ131772 | AF015436 |  |
| *Liriodendron_chinense* |  | AF123481 | AY841593 |  |  |
| *Lithocarpus_cleistocarpus* |  | EF057117 |  | EF057114 |  |
| *Lithocarpus_glaber* |  | HQ427322 | AB060568 | AY040435 |  |
| *Lithocarpus_hancei* |  |  |  | AY040451 |  |
| *Litsea_coreana* |  | HQ427405 | HQ427263 | AF272286 |  |
| *Litsea_cubeba* |  | AF244398 | AY337734 | AB260863 |  |
| *Litsea_elongata* |  | HQ427403 | HQ427261 | DQ120606 |  |
| *Lonicera_hypoglauca* |  | HM228434 | HM228478 | FJ372916 |  |
| *Lonicera_japonica* |  | GQ997392 | HM850134 | JQ780992 |  |
| *Lonicera_macranthoides* |  | HM228448 | HM228492 | FJ372918 |  |
| *Lonicera_modesta* |  |  |  | EU240716 |  |
| *Loropetalum_chinense* |  | HQ427312 | AF061999 | GU576672 |  |
| *Lyonia_ovalifolia* |  | U61305 | AF124580 |  |  |
| *Maackia_chinensis* |  |  |  | EF457721 |  |
| *Machilus_grijsii* |  | KF569899 | KF569893 | JF976985 |  |
| *Machilus_leptophylla* |  | HM019350 | HM019490 | EF538697 |  |
| *Machilus_pauhoi* |  | HQ427418 | HM019496 | EF538695 |  |
| *Machilus_thunbergii* |  | KF569890 | KF569894 | FJ755429 |  |
| *Maesa_japonica* |  |  |  | JF708192 |  |
| *Magnolia_cylindrica* |  | HQ427420 | AY008914 |  |  |
| *Magnolia_denudata* |  | AF123465 | AY008913 | EU593545 |  |
| *Magnolia_officinalis* |  | AF548641 | AY008933 | EU593549 |  |
| *Mahonia_bealei* |  | DQ478617 | L12657 | FJ424229 |  |
| *Mallotus_japonicus* |  | AB268027 | AY794934 |  |  |
| *Mallotus_repandus* |  | EF582678 | GU441787 | DQ866617 |  |
| *Malus_hupehensis* |  | AF309179 | JQ391346 | JQ392455 |  |
| *Malus_leiocalyca* |  | HQ427351 | HQ427202 |  |  |
| *Manglietia_fordiana* |  | AY952412 | L12658 |  |  |
| *Melastoma_dodecandrum* |  |  | GQ436727 | GQ265883 |  |
| *Melia_azedarach* |  | EF489117 | AY128234 | AY695595 |  |
| *Meliosma_flexuosa* |  | HQ427361 | HQ427214 |  |  |
| *Meliosma_oldhamii* |  | HQ427360 | HQ427213 |  |  |
| *Meliosma_rigida* |  | HQ415309 | HQ415132 |  |  |
| *Michelia_maudiae* |  | HQ415276 | HQ415093 | EU593553 |  |
| *Michelia_skinneriana* |  | HQ427417 | HQ427275 |  |  |
| *Microtropis_fokienensis* |  | HQ393848 |  | HQ393683 |  |
| *Millettia_dielsiana* | syn. *Callerya cinerea* |  | GQ436360 | FJ980295 |  |
| *Millettia_reticulata* | syn. *Callerya reticulata* | AF142733 |  | AF467031 |  |
| *Morus_alba* |  | AB038183 | L01933 | JN407493 |  |
| *Morus_australis* |  | GU145559 | GU145573 | AY345152 |  |
| *Morus_cathayana* |  | GU145565 | GU145579 | AM042001 |  |
| *Mussaenda_shikokiana* |  |  |  | AJ846854 |  |
| *Myrica_rubra* | syn. *Morella rubra* | HQ427396 | HQ427253 | AJ626784 |  |
| *Myrsine_stolonifera* | *Myrsine_retusa* | HM850887 | HM850193 |  |  |
| *Neolitsea_aurata* |  | HM019358 | HM019498 | JF977135 |  |
| *Nyssa_sinensis* |  | JF308675 | JF308651 | EU734444 |  |
| *Orixa_japonica* |  | EF489106 |  | HM851496 |  |
| *Ormosia_henryi* |  | HM049514 |  |  |  |
| *Osbeckia_chinensis* |  |  | AF215525 |  |  |
| *Osmanthus_cooperi* |  | EU669875 | HQ427188 | EF362772 |  |
| *Osmanthus_fragrans* |  | FM208253 |  | EU314904 |  |
| *Osmanthus_matsumuranus* |  | EU409435 |  | EF362770 |  |
| *Persea_grijsii* |  | AJ247180 |  |  |  |
| *Pertusadina_hainanensis* |  | HQ427346 | AJ347002 | AJ346892 |  |
| *Philadelphus_brachybotrys* | *Philadelphus pekinensis* | GU217268 |  |  |  |
| *Phoebe_bournei* |  | HM019369 | HM019509 | EF538706 |  |
| *Phoebe_sheareri* |  | HQ427400 | HM019513 | FM957848 |  |
| *Photinia_beauverdiana* |  | HQ427353 | HQ427204 | JQ392492 |  |
| *Photinia_glabra* |  | HQ427354 | HQ427205 | FJ796905 |  |
| *Photinia_parvifolia* |  | HQ427355 | HQ427206 | GQ368497 |  |
| *Photinia_serrulata* | syn. *Photinia serratifolia* | AF288111 | GQ436594 | GQ368486 |  |
| *Photinia_villosa* |  |  |  | FJ810016 |  |
| *Phyllanthus_glaucus* |  |  | AY765271 | HM106990 |  |
| *Phyllanthus_urinaria* |  |  | AY765268 | AY936735 |  |
| *Picrasma_quassioides* |  | HQ427327 | EU043008 | GQ434548 |  |
| *Pieris_formosa* |  | U61303 | AF124581 | EU547690 |  |
| *Pieris_japonica* |  | AB206598 | AB206589 | EU547692 |  |
| *Pieris_taiwanensis* |  | AB206599 | AB206593 |  |  |
| *Pinus_massoniana* |  | DQ353716 | DQ353732 |  |  |
| *Pinus_taiwanensis* |  | AB161016 | DQ156493 |  |  |
| *Pistacia_chinensis* |  |  | FN599457 | EF193079 |  |
| *Pittosporum_illicioides* |  | HQ427307 | HQ427157 |  |  |
| *Platycarya_strobilacea* |  | HQ427308 | AY263933 | AF303808 |  |
| *Pleioblastus_amarus* | *Arundinaria tecta* | EF125165 | AJ746179 | HQ292267 |  |
| *Podocarpus_macrophyllus* |  | AF228111 | AF249616 |  |  |
| *Podocarpus_nagi* | syn. *Nageia nagi* | AB644449 | AB644468 |  |  |
| *Polygala_arillata* |  |  | AM234210 |  |  |
| *Populus_adenopoda* | *Populus tremula* | AJ506086 | AJ418827 |  |  |
| *Pourthiaea_hirsuta* |  |  |  | GQ368494 |  |
| *Premna_microphylla* |  | HQ427331 | U28883 |  |  |
| *Prunus_discoidea* |  |  | HQ427208 |  |  |
| *Prunus_mume* |  | JF955822 | AF411491 | JF978116 |  |
| *Prunus_persica* |  | AF288117 | AF411493 | JF978127 |  |
| *Prunus_phaeosticta* |  | HQ415272 | HQ415089 | EU669095 |  |
| *Prunus_salicina* |  |  | AF411494 | AF318725 |  |
| *Prunus_schneideriana* |  | HQ427356 | HQ427209 | EU370928 |  |
| *Prunus_serrulata* |  | GU363780 | AF411487 | AF318721 |  |
| *Prunus_spinulosa* |  | HQ427357 | AF411503 | AF411513 |  |
| *Prunus_undulata* |  |  |  | EU669108 |  |
| *Pseudolarix_kaempferi* |  | AB019866 | X58782 |  |  |
| *Pterocarya_insignis* | syn. *Pterocarya macroptera* |  |  | AF303814 |  |
| *Pterocarya_stenoptera* |  | AF118042 |  | AF179587 |  |
| *Pyrus_calleryana* |  |  | JQ391379 | JQ392478 |  |
| *Quercus_acutissima* |  | AB060069 | AB060578 | AF098428 |  |
| *Quercus_fabri* |  |  |  | HE591366 |  |
| *Quercus_myrsinifolia* |  | AB060063 | AB060572 | AF098414 |  |
| *Quercus_phillyraeoides* |  | HQ427324 | AB060573 | AY040462 |  |
| *Quercus_serrata* |  | AB060067 | AB060576 |  |  |
| *Quercus_variabilis* |  | AB060065 | AB060574 | AY040463 |  |
| *Randia_cochinchinensis* | syn. *Aidia cochinchinensis* | HQ427347 | HQ427198 |  |  |
| *Rhamnella_franguloides* |  |  | AJ3900271 | AY626454 |  |
| *Rhamnus_crenata* |  | HQ427385 | HQ427242 | AY626443 |  |
| *Rhamnus_utilis* |  | JF317432 | JF317492 |  |  |
| *Rhaphiolepis_indica* |  | HQ427352 | HQ427203 | JQ392494 |  |
| *Rhododendron_fortunei* |  | AF454850 | HQ706905 | AF393407 |  |
| *Rhododendron_latoucheae* |  | HQ427298 | HQ427145 |  |  |
| *Rhododendron_mariesii* |  | AF454860 | HQ427147 | AF297202 |  |
| *Rhododendron_ovatum* |  | U61330 | HQ427144 | JF978354 |  |
| *Rhododendron_simiarum* |  |  | HQ706935 | HQ707070 |  |
| *Rhododendron_simsii* |  | HQ427299 | GQ997829 | JF978401 |  |
| *Rhus_chinensis* |  |  | FN599458 | EF682845 |  |
| *Rhus_hypoleuca* |  | HQ427342 |  |  |  |
| *Rosa_bracteata* |  | HM490026 |  |  |  |
| *Rosa_cymosa* |  | AB039317 |  | HM593924 |  |
| *Rosa_henryi* |  | AB039310 |  | AB038454 |  |
| *Rosa_laevigata* |  | AB011997 | GU363797 | JN407516 |  |
| *Rosa_multiflora* |  | AB039304 | GQ436573 | HM593923 |  |
| *Rosa_rubus* |  | FJ472525 |  | FJ416660 |  |
| *Rubus_amphidasys* |  |  |  | AY083367 |  |
| *Rubus_buergeri* |  |  |  | FJ472903 |  |
| *Rubus_chingii* |  | HQ427358 | HQ427211 |  |  |
| *Rubus_corchorifolius* |  |  |  | JF708203 |  |
| *Rubus_coreanus* |  |  |  | FJ472906 |  |
| *Rubus_hirsutus* |  | GU363753 | GU363792 | FJ472891 |  |
| *Rubus_hunanensis* |  |  |  | FJ472902 |  |
| *Rubus_irenaeus* |  |  |  | EF034131 |  |
| *Rubus_lambertianus* |  |  |  | FJ472904 |  |
| *Rubus_parvifolius* |  | AB073699 | GU363802 | JN407526 |  |
| *Rubus_pungens* |  |  |  | FJ472893 |  |
| *Rubus_reflexus* |  | JN407197 | JN407362 | JN407520 |  |
| *Rubus_swinhoei* |  |  |  | EF034143 |  |
| *Rubus_tephrodes* |  |  |  | EF034144 |  |
| *Rubus_trianthus* |  |  |  | AY083366 |  |
| *Sabia_campanulata* |  |  | AM183414 |  |  |
| *Sabia_japonica* |  | AM396512 |  |  |  |
| *Sabia_swinhoei* |  | GU266603 | FJ626616 |  |  |
| *Sageretia_thea* |  |  | AJ2257851 | AY626453 |  |
| *Salix_babylonica* |  | AJ849593 | FJ788588 |  |  |
| *Sambucus_williamsii* |  |  |  | JN040994 |  |
| *Sapindus_mukorossi* |  |  | FN599461 |  |  |
| *Sapium_discolor* | syn. *Triadica cochinchinensis* | HQ415366 | HQ415199 | JF733770 |  |
| *Sapium_japonicum* | syn. *Neoshirakia japonica* |  | AY794856 |  |  |
| *Sapium_sebiferum* | syn. *Triadica sebifera* | GU135113 | AY794859 | GU441830 |  |
| *Sassafras_tzumu* |  | AF244391 | HM019516 | GU082375 |  |
| *Schima_superba* |  | AJ429306 | Z80208 | HM100443 |  |
| *Schoepfia_jasminodora* |  | HQ415321 | HQ415146 |  |  |
| *Securinega_suffruticosa* | *Securinega capuronii* |  | AY663621 |  |  |
| *Serissa_foetida* | syn. *Serissa serissoides* |  | Z68822 | FJ980385 |  |
| *Skimmia_reevesiana* |  | FN668822 | FN599464 |  |  |
| *Sloanea_sinensis* |  |  | HQ427152 |  |  |
| *Sorbus_alnifolia* | syn. *Aria alnifolia* | DQ860451 |  | FJ810006 |  |
| *Sorbus_dunnii* | syn. *Aria dunnii* |  |  | GQ368505 |  |
| *Sorbus_folgneri* |  | HQ427359 | HQ427212 |  |  |
| *Sorbus_hemsleyi* |  |  |  | FJ810010 |  |
| *Spiraea_blumei* |  | JQ041791 |  | JQ041773 |  |
| *Spiraea_cantoniensis* |  | AF288127 |  | DQ897609 |  |
| *Spiraea_chinensis* |  | JQ041792 |  | JQ041774 |  |
| *Spiraea_japonica* |  |  |  | DQ897617 |  |
| *Spiraea_prunifolia* |  | JQ041787 |  | DQ897623 |  |
| *Spiraea_vanhouttei* |  |  | L11206 | U16205 |  |
| *Stachyurus_chinensis* |  | AM396501 | JF944501 | DQ307102 |  |
| *Stauntonia_hexaphylla* |  | FJ626517 | L37922 | AY029784 |  |
| *Stephanandra_chinensis* |  | AF288128 |  | AF487153 |  |
| *Stewartia_sinensis* |  | AF380106 | AF380061 | AY070322 |  |
| *Styrax_calvescens* |  |  |  | AF327468 |  |
| *Styrax_dasyanthus* |  | HQ427280 | HQ427123 | AF327469 |  |
| *Styrax_faberi* |  |  |  | AF327484 |  |
| *Styrax_japonicus* |  |  |  | AF327465 |  |
| *Styrax_odoratissimus* |  | HQ427282 | HQ427125 | AF327460 |  |
| *Styrax_suberifolius* |  | HQ427281 | HQ427124 | AF327493 |  |
| *Styrax wuyuanensis* | added manually to ML tree |  |  |  |  |
| *Symplocos_anomala* |  | AY679808 | HQ427233 | AY336291 |  |
| *Symplocos_chinensis* |  | AY336341 |  | AF396229 |  |
| *Symplocos_heishanensis* |  |  |  | AY630642 |  |
| *Symplocos_lancifolia* |  | HQ415339 | HQ415167 | AB114887 |  |
| *Symplocos_laurina* |  | AY336368 |  | AY336318 |  |
| *Symplocos_oblongifolia* | added manually to ML tree |  |  |  |  |
| *Symplocos_paniculata* |  | AF440433 | Z83139 | AY336263 |  |
| *Symplocos_phyllocalyx* |  | AY336357 |  | AY336293 |  |
| *Symplocos_setchuensis* |  | AY336359 | HQ427235 | AY336294 |  |
| *Symplocos_stellaris* |  | HQ427379 | HQ427236 | AY336329 |  |
| *Symplocos_sumuntia* |  | HQ427377 |  | AY336322 |  |
| *Syzygium_buxifolium* |  | HQ415314 | HQ427244 | EF026624 |  |
| *Tarenna_mollissima* |  | HQ415401 |  |  |  |
| *Taxodium_distichum* |  | JQ512482 | AF119185 |  |  |
| *Taxus_chinensis* |  |  | AY450856 |  |  |
| *Ternstroemia_gymnanthera* |  | AF380109 | AF421106 | HM061522 |  |
| *Tilia_endochrysea* |  | HQ427306 | HQ427156 |  |  |
| *Toona_ciliata* |  |  |  | FJ462489 |  |
| *Toona_sinensis* |  | JN680343 | JN654542 | FJ462490 |  |
| *Torreya_grandis* |  | AF228108 | DQ478794 |  |  |
| *Toxicodendron_succedaneum* |  | HQ427343 | AY510144 | FJ945957 |  |
| *Toxicodendron_sylvestre* |  | HQ415319 | AY510145 | FJ945938 |  |
| *Toxicodendron_trichocarpum* |  |  | AY510143 | FJ945927 |  |
| *Trachycarpus_fortunei* |  | HQ720315 | AY012460 |  |  |
| *Trema_cannabina* | *Trema micrantha* | GQ982115 | AF062004 | AY635571 |  |
| *Tricalysia_dubia* | *Diplospora dubia* | HQ427350 | HQ427201 |  |  |
| *Tutcheria_microcarpa* |  | HQ427376 | HQ427231 | AF456277 |  |
| *Ulmus_parvifolia* |  | AF345321 | D86316 |  |  |
| *Vaccinium_bracteatum* |  | AB623177 | KF569892 |  |  |
| *Vaccinium_carlesii* |  |  | KF569891 |  |  |
| *Vaccinium_japonicum* | syn. *Vaccinium erythrocarpum* | AF419710 |  | AF419781 |  |
| *Vaccinium_mandarinorum* | added manually to ML tree |  |  |  |  |
| *Vernicia_fordii* |  | GU135095 | GU135180 | |  |
| *Vernicia_montana* |  | AB268057 | AY794899 | |  |
| *Viburnum_dilatatum* |  | HQ591575 | HQ591719 | JF979005 |  |
| *Viburnum_erosum* |  | HQ427362 | HQ427216 | JF979007 |  |
| *Viburnum_fordiae* |  | JF956802 | JF944784 |  |  |
| *Viburnum_plicatum* |  | HQ591613 | HQ591754 | AY265143 |  |
| *Viburnum_propinquum* |  | HQ591614 | HQ591755 | EF462987 |  |
| *Viburnum_sempervirens* |  | HQ427363 | HQ427217 | HQ591976 |  |
| *Viburnum_setigerum* |  | EF490251 | GQ248708 | HQ591977 |  |
| *Viburnum_sympodiale* |  | HQ591630 | HQ591770 | EF462988 |  |
| *Vitex_negundo* |  | AB284176 | JQ322525 | FM200123 |  |
| *Weigela_japonica* |  | HQ427364 | HQ427218 | AF078716 |  |
| *Wikstroemia_indica* |  | HQ415322 | HQ415147 |  |  |
| *Wikstroemia_monnula* |  |  | HQ427215 |  |  |
| *Xylosma_japonica* | syn. *Xylosma congesta* | AB233834 | AB233938 | DQ521290 |  |
| *Zanthoxylum_ailanthoides* |  |  | FN599470 | HM851475 |  |
| *Zanthoxylum_armatum* |  |  | GQ436751 | HM851465 |  |
| *Zanthoxylum_austrosinense* |  |  |  | HM851488 |  |
| *Zanthoxylum_simulans* |  | EF489100 |  | HM851466 |  |
| *Zelkova_schneideriana* |  | AF345328 |  | AJ622867 |  |
| *Zelkova_serrata* |  |  | AF206835 | AJ622877 |  |

**Table S5** Age constraints for nodes used to create the ultrametric tree.

| **Clade** | **Node defined by MRCA to** | **Calibration type** | **Age [ma]** | **Reference** |
| --- | --- | --- | --- | --- |
| Seed plants | *Taxodium distichum - Abutilon theophrasti* | max | 385 | (Gerrienne et al. 2004) |
| Gymnosperms | *Pseudolarix kaempferi - Taxodium distichum* | min | 318 | (Renner 2009) |
| Cupressaceae | *Cunninghamia lanceolata - Taxodium distichum* | min | 90 | (LePage 2003) |
| Pinaceae | *Pseudolarix kaempferi - Pinus massoniana* | min | 90 | (Gandolfo et al. 2001) |
| Angiosperms | *Pleioblastus amarus - Abutilon theophrasti* | max | 130 | (Hughes and McDougall 1987, Hughes et al. 1991) |
| Laurales | *Chimonanthus salicifolius - Litsea cubeba* | min | 108.8 | (Crane et al. 1994) |
| Eudicots | *Holboellia coriacea - Abutilon theophrasti* | fixed | 125 | (Hughes and McDougall 1990) |
| Ranunculales | *Holboellia coriacea - Mahonia bealei* | min | 91 | (Knobloch and Mai 1986) |
| Berberidaceae | *Berberis soulieana - Mahonia bealei* | min | 33.9 | (Manchester 1999) |
| Hamamelidaceae | *Liquidambar acalycina - Corylopsis sinensis* | min | 83.5 | (Magallon-Puebla et al. 1996, Magallón et al. 2001) |
| Fabales | *Polygala arillata - Albizia kalkora* | min | 60 | (Lavin et al. 2005) |
| Malpighiales | *Vernicia fordii - Phyllanthus urinaria* | min | 89.3 | (Crepet and Nixon 1998) |
| Salicaceae | *Idesia polycarpa - Populus adenopoda* | min | 48 | (Boucher et al. 2003) |
| Fagales | *Quercus serrata - Juglans cathayensis* | min | 93.5 | (Pacltová 1966, Batten 1981, Kedves 1989) |
| Juglandaceae | *Cyclocarya paliurus - Juglans cathayensis* | min | 55.8 | (Crane et al. 1990) |
| Rosaceae | *Rosa cymosa - Prunus pseudocerasus* | min | 37.2 | (Manchester 1999) |
| Ulmaceae | *Ulmus parvifolia - Zelkova schneideriana* | min | 33.9 | (Manchester 1999) |
| Rutaceae-Meliaceae | *Melia azedarach - Skimmia japonica* | min | 50 | (Corbett and Manchester 2004) |
| Myrtales | *Melastoma dodecandrum - Syzygium buxifolium* | min | 60 | (Pigg et al. 1993) |
| Ericales | *Actinidia melanandra - Ardisia crenata* | min | 89.6 | (Nixon and Crepet 1993) |
| Actinidiaceae (stem node) | *Actinidia melanandra - Rhododendron latoucheae* | min | 77.05 | (Schenk and Hufford 2010) |
| Cornaceae | *Alangium kurzii - Cornus kousa* | min | 55.8 | (Manchester 1999) |
| Nyssaceae | *Camptotheca acuminata - Nyssa sinensis* | min | 33.9 | (Manchester 1999) |
| Hydrangeaceae | *Deutzia glauca - Hydrangea strigosa* | min | 37.2 | (Manchester 1999) |
| Cornales | *Camptotheca acuminata - Cornus kousa* | min | 89 | (Schenk and Hufford 2010) |
| Oleaceae | *Osmanthus matsumuranus - Fraxinus chinensis* | min | 33.9 | (Manchester 1999) |
| Dipsacales | *Viburnum sympodiale - Lonicera modesta* | min | 33.9 | (Manchester and Donoghue 1995) |
| Apiales | *Pittosporum illicioides - Dendropanax dentiger* | min | 37.2 | (Manchester 1999) |

**Table S6** Loadings and percentage of total variation explained of the first six principal components (PCs) of a PCA on the eleven environmental variables. The first two PCs correspond to variation in soil moisture and light, respectively. See Fig. S7 for PCA biplot.

|  | **PC1** | **PC2** | **PC3** | **PC4** | **PC5** | **PC6** |
| --- | --- | --- | --- | --- | --- | --- |
| **Elevation** | 0.8 | 0.47 | -0.09 | 0.5 | -0.08 | 0.47 |
| **Aspect_Eastness** | -0.28 | -0.65 | 0.51 | -0.06 | 0.67 | 0.54 |
| **Aspect_Northness** | 0.26 | -0.48 | 0.83 | 0.17 | -0.54 | 0.22 |
| **Slope** | 0.49 | -0.38 | 0.7 | -0.65 | 0.04 | -0.29 |
| **Light (PAR)** | -0.08 | -1.03 | -0.09 | 0.5 | 0.2 | -0.29 |
| **Red/far-red** | 0.07 | 1.07 | 0.23 | -0.36 | 0 | 0.11 |
| **Soil moisture** | 1.14 | -0.23 | -0.15 | 0.03 | -0.05 | -0.1 |
| **pH** | -0.63 | -0.38 | -0.58 | -0.58 | -0.12 | 0.29 |
| **Soil C/N** | -0.49 | 0.55 | 0.83 | 0.2 | 0.08 | -0.13 |
| **N mineral** | 0.82 | 0.45 | -0.09 | -0.03 | 0.64 | -0.13 |
| **P total** | 1.02 | -0.48 | -0.07 | -0.34 | -0.12 | 0.16 |
| **Cumulative variance explained (in %)** | 27.7 | 52.3 | 67.6 | 76.9 | 84.2 | 89.5 |

**Table S7** Phylogenetic signal (Blomberg's *K*, Pagel's *λ* and Abouheif/Moran's *I*) in each of the six traits.Values of Blomberg's *K* and Pagel's *λ* equal to one correspond to a Brownian motion model of trait evolution, while values of *K* or *λ* close to zero indicate no phylogenetic signal. Unlike *K* and *λ,* Abouheif/Moran's *I* is a measure of phylogenetic autocorrelation and is not based on an evolutionary model. *P*-values for the *K-* and *I-* statistics were obtained by randomly shuffling (999 times) the tips on the phyogeny. *P*-values for Pagel's *λ* were obtained based on likelihood-ratio tests.

|  | **Leaf area** | **SLA** | **Leaf N** | **Leaf P** | **Wood density** | **Height** |
| --- | --- | --- | --- | --- | --- | --- |
| Blomberg’s *K* | 0.902 | 0.385 | 0.726 | 0.576 | 0.534 | 0.46 |
| *P* | < 0.001 | 0.023 | < 0.001 | < 0.001 | < 0.001 | 0.005 |
| Pagel’s *λ* | 0.991 | 0.45 | 0.902 | 0.596 | 0.612 | 0.479 |
| *P* | < 0.001 | < 0.001 | < 0.001 | < 0.001 | < 0.001 | < 0.001 |
| Abouheif/Moran’s *I* | 0.266 | 0.22 | 0.32 | 0.246 | 0.248 | 0.125 |
| *P* | < 0.001 | < 0.001 | < 0.001 | < 0.001 | < 0.001 | 0.028 |

**Table S8** Nodes with significantly more taxa than expected within a particular plot (CSP). Columns correspond to successional stage (Stage 1-5), Plot ID (see also Fig. 1c), node name (see Fig. S6), and ranks in the null distribution across 999 randomization runs, shuffling the tips in the phylogeny. Only nodes that are within the upper 2.5-percentile of the null distribution are listed. Highlighted (for illustration purposes) are the most significant nodes, associated with the plot pairs that had the highest levels of phylogenetic turnover at the two late successional stages (red: stage 4, CSPs 5 & 11; blue: stage 5, CSPs 4 & 12) (see also Fig. 1c and Fig. S6).

**Successional Stage Plot ID (CSP) Node name Rank**

1 16 N22 990

1 16 N42 975

1 16 N44 989

1 19 N42 980

1 19 N44 992

1 20 N39 994

1 20 N40 985

1 22 N44 989

1 26 N86 983

1 26 N92 986

2 23 N92 974

2 24 N22 995

2 24 N23 982

3 1 N8 976

3 3 N44 985

3 3 N78 991

3 6 N20 981

3 6 N21 985

3 6 N22 999

3 6 N23 987

3 6 N24 992

3 6 N26 983

3 6 N44 974

3 7 N22 999

3 7 N24 990

3 7 N25 999

3 7 N42 989

3 7 N44 998

3 7 N45 983

3 8 N22 974

3 8 N92 974

3 8 N93 993

3 8 N94 984

4 11 N22 995

4 11 N23 982

4 11 N29 988

4 11 N30 993

4 11 N39 999

4 11 N40 988

4 13 N24 975

4 5 N22 988

4 5 N42 995

4 5 N44 999

4 5 N48 991

4 10 N20 983

4 10 N22 999

4 10 N23 999

4 10 N24 990

4 10 N25 979

4 10 N29 994

4 10 N30 988

4 10 N33 997

4 18 N22 996

4 18 N23 997

4 18 N24 977

5 4 N58 980

5 12 N24 978

5 14 N77 999

5 14 N78 997

5 14 N79 986

**Methods S1** We gathered sequence information, i.e. *mat*K, *rbc*L and the ITS region including the 5.8s gene for all woody species from Gutianshan National Nature Reserve (Lou & Li, 1998) or closely related species available in GenBank (<http://www.ncbi.nlm.nih.gov/genbank/>, accessed between May and June 2012). For some species of the CSPs, *mat*K and *rbc*L were sequenced using standard barcoding protocols (Fazekas *et al.*, 2012) (Accession numbers: KF569888-KF569899, Table S4). All sequences were aligned separately for the different markers using MAFFT v6 (Katoh *et al.*, 2002). Sequences for *mat*K and *rbc*L were aligned with the ‘Auto’ option in the online version of the program (<http://mafft.cbrc.jp/alignment/server/>). The ITS region was aligned with the ‘Q-INS-I’ option considering secondary structure of RNA using the MAFFT application at Bioportal (https://www.bioportal.uio.no/, Kumar *et al.*, 2009)). Aligned sequences were concatenated for each species resulting in a total alignment of 3521 nucleotide positions. A phylogenetic tree was inferred using a Maximum Likelihood (ML) method implemented in PhyML (Guindon & Gascuel, 2003). For ML inference, the best fitting model (GTR+I+G) selected by Modeltest (Posada and Crandall 1998) was applied with the following options: tree topology search operation: best of NNI and SPR search, number of substitution rate categories =6, all other parameters were estimated (Gamma Distribution Parameter Alpha, Proportion of Invariable Sites, Transition/Transversion Ratio).

Species occurring in the CSPs but without sequence information available (Table S4) were added manually to the obtained ML tree by the following procedure. *Acer cordatum* was added within *Acer* as a polytomy to the most recent common ancestor (MRCA) of a monophyletic clade formed by other members of *Acer* sect. *Palmata* (i.e. *A*. *elegantulum*, *A*. *wilsonii*, *A*. *olivaceum*). Its branch length was defined as the average distance from the MRCA of that clade to the tips. *Styrax wuyuanensis*, *Symplocos oblongifolia* and *Vaccinium mandarinorum* were added similarily as polytomy emerging from the MRCA for all other members of the respective genus included, with branch lengths equalling the average branch length from that MRCA to the tips of congeners.

Using the ML topology and branch lengths an ultrametric tree was created by non-parametric rate smoothing (nprs) as implemented in r8s (Sanderson, 1997). Absolute node ages were obtained using 27 published fossils or dates as age constrains. A fixed age of 125 million years was applied to the crown node of the Eudicots (Table S5).

**Methods S4** We tested, based on 100 simulation runs, whether differences in the number of plots (communities) among stages affect the estimates or phylogenetic turnover (Π_ST_ and B_ST_) using the following procedure: in each simulation run we (i) generated 10 communities with 10 species each, and 20 species in total, (ii) calculated Π_ST_ (or B_ST_) based on different numbers of plots (3-10 plots) and assessed the Pearson-correlation between Π_ST_ (or B_ST_) and the number of plots, and (iii) tested (using a one sample t-test) whether the mean correlation obtained from the 100 simulations significantly differed from zero. Calculations of Π_ST_ (or B_ST_) were based on a random Yule (pure-birth) tree for 20 tips [R-package 'phytools' (Revell, 2012)]. We found that the mean correlation between Π_ST_ (and B_ST_) and the number plots was close to zero, indicating that there is no intrinsic correlation between the phylogenetic turnover estimates used in our study and the number of plots.

**Methods S5** Because non-random phylogenetic structure at the plot scale may simply reflect non-random pattern in overall species frequencies (or abundances) across the phylogeny (Mouquet *et al.*, 2012), we tested for phylogenetic signal in species occurrences as well as abundances at the scale of the whole data set using the APD (abundance phylogenetic deviation) index proposed by Hardy (2008). There was no phylogenetic signal in overall species' occurrence frequencies or abundances in our study (APD = 0.014, *P* = 0.056 and APD = 0.053, *P* = 0.996), so there was no need to implement a null model that restricts permutations to species with similar occurrence frequencies (or abundances).

**References**

**Batten DJ. 1981.** Stratigraphic, palaeogeographic and evolutionary significance of late cretaceous and early tertiary normapolles pollen*. Review of Palaeobotany and Palynology* **35**: 125–137.

**Boucher LD, Manchester SR, Judd WS. 2003.** An extinct genus of Salicaceae based on twigs with attached flowers, fruits, and foliage from the Eocene Green River Formation of Utah and Colorado, USA. *American Journal of Botany* **90**: 1389–1399.

**Corbett SL, Manchester SR. 2004.** Phytogeography and fossil history of Ailanthus (Simaroubaceae). *International Journal of Plant Sciences* **165**: 671–690.

**Crane PR, Friis EM, Pedersen KR. 1994.** Palaeobotanical evidence on the early radiation of magnoliid angiosperms. *Plant Systematics and Evolution - Supplementa* **8**: 51–72.

**Crane PR, Manchester SR, Dilcher DL. 1990.** A preliminary survey of fossil leaves and well-preserved reproductive structures from the Sentinel Butte Formation (Paleocene) near Almont, North Dakota. *Fieldiana. Geology* **20**: 1–63.

**Crepet WL, Nixon KC. 1998.** Fossil Clusiaceae from the Late Cretaceous (Turonian) of New Jersey and implications regarding the history of bee pollination. *American Journal of Botany* **85**: 1122–1133.

**Fazekas AJ, Kuzmina ML, Newmaster SG, Hollingsworth PM. 2012.** DNA Barcoding Methods for Land Plants In: DNA Barcodes: Methods and protocols (eds. Kress WJ, Erickson DL), pp. 223–252. Humana Press, New York.

**Gandolfo MA, Nixon KC, Crepet WL. 2001.** Turonian Pinaceae of the Raritan Formation, New Jersey. *Plant Systematics and Evolution* **226**: 187–203.

**Gerrienne P, Meyer-Berthaud B, Fairon-Demaret M, Streel M and Steemans P. 2004.** Runcaria, a Middle Devonian Seed Plant Precursor. *Science* **306**: 856–858.

**Guindon S, Gascuel O. 2003.** A Simple, Fast, and Accurate Algorithm to Estimate Large Phylogenies by Maximum Likelihood. *Systematic Biology* **52**: 696–704.

**Hardy OJ. 2008.** Testing the spatial phylogenetic structure of local communities: statistical performances of different null models and test statistics on a locally neutral community. *Journal of Ecology* **96**: 914–926.

**Hughes NF, McDougall AB. 1987.** Records of angiospermid pollen entry into the english early cretaceous succession. *Review of Palaeobotany and Palynology* **50**: 255–272.

**Hughes NF, McDougall AB. 1990.** Barremian-Aptian angiospermid pollen records from southern England. *Review of Palaeobotany and Palynology* **65**: 145–151.

**Hughes NF, McDougall AB, Chapman JL. 1991.** Exceptional new record of Cretaceous Hauterivian angiospermid pollen from Southern England. *Journal of Micropalaeontology* **10**: 75–82.

**Katoh K, Misawa K, Kuma K, Miyata, T. 2002.** MAFFT: a novel method for rapid multiple sequence alignment based on fast Fourier transform. *Nucleic Acids Research* **30**: 3059–3066.

**Kedves M. 1989.** Evolution of the Normapolles complex. In: Evolution, Systematics, and Fossil History of the Hamamelidae, 1-7. Systematics Association Special Volume, vol. 40B. (eds. Crane P. R, Blackmore S.). Clarendon Press, Oxford.

**Knobloch E, Mai DH. 1986.** Monographie der Früchte und Samen in der Kreide von Mitteleuropa, Praha.

**Kumar S, Skjaeveland A, Orr R, Enger P, Ruden T, Mevik B-H, Burki F, Botnen A, Shalchian-Tabrizi K. 2009.** AIR: A batch-oriented web program package for construction of supermatrices ready for phylogenomic analyses. *BMC Bioinformatics* **10**: 357.

**Lavin M, Herendeen PS, Wojciechowski MF. 2005.** Evolutionary rates analysis of Leguminosae implicates a rapid diversification of lineages during the Tertiary. *Systematic Biology* **54**: 575–594.

**LePage BA. 2003.** The evolution, biogeography and palaeoecology of the Pinaceae based on fossil and extant representatives. *Acta Horticulturae* **615**: 29–52.

**Lou LH, Li GY. 1998.** List of seed plants in Gutianshan.

**Magallon-Puebla S, Herendeen PS, Endress PK. 1996.** Allonia decandra: Floral remains of the tribe Hamamelideae (Hamamelidaceae) from Campanian strata of southeastern USA. *Plant Systematics and Evolution* **202**: 177–198.

**Magallón S, Herendeen PS, Crane P. 2001.** Androdecidua endressii gen. et sp. nov, from the Late Cretaceous of Georgia (United States): Further Floral Diversity in Hamamelidoideae (Hamamelidaceae). *International Journal of Plant Sciences* **162**: 963–983.

**Manchester SR. 1999.** Biogeographical relationships of North American tertiary floras. *Annals of the Missouri Botanical Garden* **86**: 472–522.

**Manchester SR, Donoghue MJ. 1995.** Winged fruits of Linnaeeae (Caprifoliaceae) in the Tertiary of Western North America: Diplodipelta gen. nov. *International Journal of Plant Sciences* **156**: 709–722.

**Mouquet N, Devictor V, Meynard CN, Munoz F, Bersier L-F, Chave J, Couteron P, Dalecky A, Fontaine C, Gravel D *et al.* 2012.** Ecophylogenetics: advances and perspectives. *Biological Reviews* **87**: 769–785.

**Nixon KC, Crepet WL. 1993.** Late Cretaceous fossil flowers of ericalean affinity. *American Journal of Botany* **80**: 616–623.

**Pacltová B. 1966.** Pollen grains of angiosperms in the Cenomanian Peruc Formation in Bohemia. *Palaeobotanist* **15**: 52–54.

**Pigg KB, Stockey RA, Maxwell SL. 1993.** Paleomyrtinaea, a new genus of permineralized myrtaceous fruits and seeds from the Eocene of British Columbia and Paleocene of North Dakota. *Canadian Journal of Botany* **71**: 1–9.

**Posada D, Crandall KA. 1998.** MODELTEST: testing the model of DNA substitution. *Bioinformatics* **14**: 817–818.

**Renner S. 2009.** Gymnosperms. In: The Timetree of Life (eds. Hedges SB, Kumar S), pp. 157–160. Oxford University Press, Oxford.

**Revell LJ. 2012.** phytools: an R package for phylogenetic comparative biology (and other things). *Methods in Ecology and Evolution* **3**: 217–223.

**Sanderson MJ. 1997.** A nonparametric approach to estimating divergence times in the absence of rate constancy. *Molecular Biology and Evolution* **14**: 1218–1231.

**Schenk JJ, Hufford L. 2010.** Effects of substitution models on divergence time estimates: Simulations and an empirical study of model uncertainty using Cornales. *Systematic Botany* **35**: 578–592.
